# Phylogeny Reveals Novel HipA-Homologous Kinase Families and Toxin-Antitoxin Gene Organizations

**DOI:** 10.1128/mBio.01058-21

**Published:** 2021-06-01

**Authors:** Kenn Gerdes, Rene Bærentsen, Ditlev E. Brodersen

**Affiliations:** a Centre of Excellence for Bacterial Stress Response and Persistence, Section for Functional Genomics, Department of Biology, University of Copenhagen, Copenhagen N, Denmark; b Department of Molecular Biology and Genetics, Aarhus University, Aarhus C, Denmark; Massachusetts Institute of Technology

**Keywords:** high persister A, HipB, HipS, HipT, HIRAN, Stl, GltX, TrpS, kinase

## Abstract

Toxin-antitoxin modules function in the genetic stability of mobile genetic elements, bacteriophage defense, and antibiotic tolerance. A gain-of-function mutation of the Escherichia coli K-12 *hipBA* module can induce antibiotic tolerance in a subpopulation of bacterial cells, a phenomenon known as persistence. HipA is a Ser/Thr kinase that phosphorylates and inactivates glutamyl tRNA synthetase, inhibiting cellular translation and inducing the stringent response. Additional characterized HipA homologues include HipT from pathogenic E. coli O127 and YjjJ of E. coli K-12, which are encoded by tricistronic *hipBST* and monocistronic operons, respectively. The apparent diversity of HipA homologues in bacterial genomes inspired us to investigate overall phylogeny. Here, we present a comprehensive phylogenetic analysis of the Hip kinases in bacteria and archaea that expands on this diversity by revealing seven novel kinase families. Kinases of one family, encoded by monocistronic operons, consist of an N-terminal core kinase domain, a HipS-like domain, and a HIRAN (HIP116 Rad5p N-terminal) domain. HIRAN domains bind single- or double-stranded DNA ends. Moreover, five types of bicistronic kinase operons encode putative antitoxins with HipS-HIRAN, HipS, γδ-resolvase, or Stl repressor-like domains. Finally, our analysis indicates that reversion of *hipBA* gene order happened independently several times during evolution.

Prokaryotic toxin-antitoxin (TA) modules were discovered due to their ability to stabilize plasmids by killing of plasmid-free cells by a mechanism known as postsegregational killing (1, 2). The mechanism relies on stable protein toxins that are inhibited either by unstable antitoxin RNAs (type I and III TAs) or unstable antitoxin proteins (type II TAs) as long as the plasmid remains in the cell. If, on the other hand, the plasmid is lost, degradation of antitoxin leads to toxin activation and hence, death of the plasmid-free cell. Since their discovery on plasmids, TAs have been identified on a wide range of bacterial and archaeal chromosomes as well (3–5), often in multiple or even large numbers (5–9). For example, Mycobacterium tuberculosis carries genes that encode ∼70 type II TA modules (7, 10, 11), while the plant symbiont Sinorhizobium meliloti contains more than 100 (12). The biological functions of chromosome-encoded TAs are debated, but experimental evidence supports at least three roles that are not mutually exclusive: (i) genetic stabilization of chromosome segments or entire chromosomes (13–16), (ii) antiphage defense by abortive infection (17, 18), and (iii) antibiotic tolerance (19–22). Intriguingly, it was recently discovered that bacterial retrons encode a type of three-component TAs that can function as antiphage defense systems, thus supporting the notion that a major function of TAs may in fact be to curb or control bacteriophage infection (23–26). This idea is consistent with the recent observation that environmental or nutritional stress in general does not activate type II TA-encoded toxins (27).

There is evidence supporting that some TAs induce antibiotic tolerance or persistence in bacteria. Persistence is a phenomenon found in all bacteria tested (19, 28–30) and is operationally defined as the subpopulation of a bacterial cells that survive for an extended period of time in the presence of inhibitory concentrations of antibiotics (31). Common to persistence mechanisms is that the phenotype is a stochastic phenomenon and expressed by only a fraction of the cell population at any given time (31, 32). Importantly, persistence is believed to contribute to the recalcitrance of bacterial infections and may thus pose a significant medical problem (30, 33–35). At the mechanistic level, persisters are slow-growing cells that display increased survival rates in the presence of antibiotics (32, 36, 37). In addition, this also buys the bacterial population time to develop true antibiotic resistance (38). Apart from a reduced growth rate, persister cells can also arise from expression of high levels of factors that counter the effects of antibiotics in a small subpopulation of cells (39, 40).

*hipA* (high persister gene A) of Escherichia coli K-12 was the first gene found to be associated with increased persistence based on the identification of the gain-of-function allele *hipA7* in a strain exhibiting increased tolerance toward penicillin (41). The mutant allele, later also found in clinical isolates of uropathogenic E. coli (28), exhibits a 100- to 1,000-fold increased level of persistence (32, 42), but even the wild-type *hipBA* module can be shown to confer a modest, but measurable, increase of persistence (28). The *hipA* toxin gene and its upstream *hipB* antitoxin gene constitute a canonical type II TA module encoding two proteins that combine to form an inactive HipBA complex, which, upon degradation of HipB, generates active HipA toxin (Fig. 1A) (43). Consequently, ectopic production of HipA in E. coli causes severe growth inhibition that can be reversed by later expression of HipB antitoxin (43). HipBA from both E. coli K-12 and Shewanella oneidensis MR-1 assemble into heterotetrameric HipA_2_B_2_ complexes (28, 44–48).

**FIG 1:**
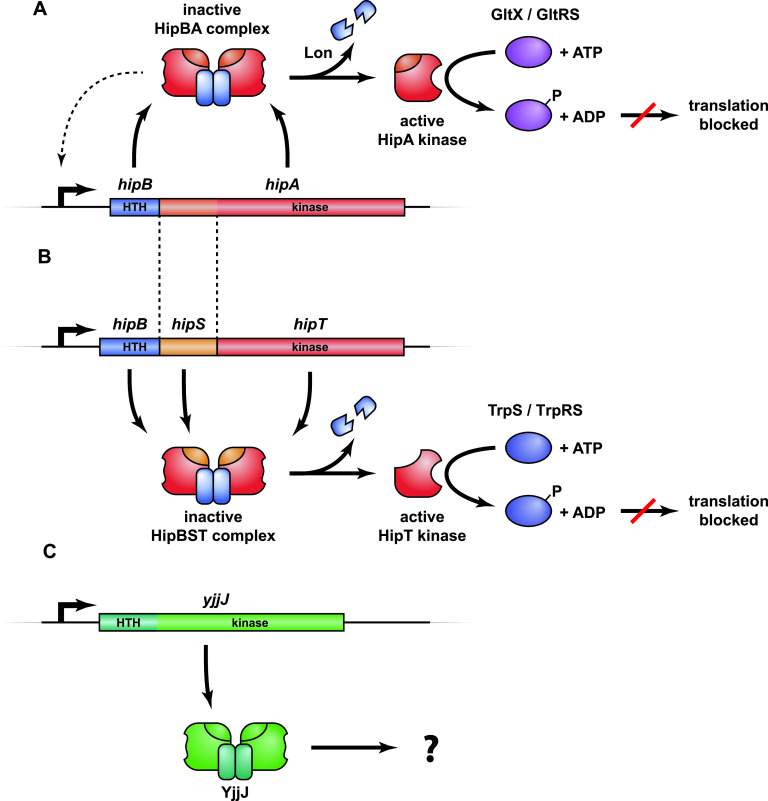
Overview of the *hipBA*, *hipBST*, and *yjjJ* operons and their protein products. (A) *hipBA* encodes the antitoxin HipB (blue) and toxin kinase HipA (red/orange) that form an inactive HipB_2_HipA_2_ complex that can bind to operators in the promoter region via HTH DNA-binding domains in HipB and thereby autoregulate transcription (dashed line) (28). Upon HipB degradation by Lon protease (88) and activation of HipA, HipA phosphorylates and inhibits glutamine tRNA synthetase (GltX/GltRS), thereby halting translation and inducing the stringent response (51, 52, 54). (B) *hipBST* encodes three proteins, HipB (blue), HipS (orange), and HipT (red) that form an inactive HipBST complex (59). HipS is homologous to the N-subdomain-1 of HipA and functions as the antitoxin that neutralizes HipT (59). Like HipB of HipBA, HipB of HipBST has an HTH domain and augments the inhibition of HipT by HipS but does not function as an antitoxin on its own. Free HipT phosphorylates and inhibits tryptophan tRNA synthetase (TrpS) and thereby halts translation in a similar fashion as HipA. (C) *yjjJ* is a single cistron operon that encodes a HipA-homologous kinase YjjJ (green) that, when overproduced, inhibits cell growth (60). YjjJ, that we coin HipH, has an HTH-domain in its N terminus that may function to bind DNA.

HipA is a so-called Hanks serine-threonine kinase (49, 50) that phosphorylates a conserved serine residue (Ser^239^) in glutamyl-tRNA synthetase (GltX) inside its bacterial host, inhibiting the enzyme and thereby aminoacylation of tRNA^Glu^ (51, 52). As a consequence, the ratio of charged to uncharged tRNA^Glu^ decreases, thus stimulating RelA-tRNA^Glu^ complexes to bind the ribosomal A site. Activation of RelA (53) on the ribosome leads to an increase in the cellular (p)ppGpp level triggering the stringent response (42, 51, 54). HipA shares its fold with human cyclin-dependent kinases and maintains all of the conserved motifs necessary for kinase activity (45). The antitoxin HipB contains a classical (Cro-like) helix-turn-helix (HTH) DNA-binding domain (45) and forms a homodimer when in complex with HipA that allows binding to palindromic operator sequences in the *hipBA* promoter region (Fig. 1A). The mechanism of toxin inhibition by HipB has not been fully elucidated, but it appears to differ from most type II systems in that the antitoxin does not interact directly with the toxin active site. Instead, the very C terminus of HipB appears to bind in a pocket on HipA, possibly regulating toxin activity (44). Finally, HipA can inactivate its own kinase activity by autophosphorylation (55), a phenomenon that has been proposed to function in resuscitation of persister cells (46, 56).

HipA homologues are present in many bacteria. For example, the chromosome of the Alphaproteobacterium Caulobacter crescentus contains three *hipBA* loci for which the encoded HipA toxins inhibit protein synthesis upon ectopic expression in all three cases (57). Like their E. coli counterpart, HipA_1_ and HipA_2_ contribute to persistence during stationary phase by phosphorylating aminoacyl-tRNA synthetases *in vivo*: HipA_2_ targets lysyl-tRNA synthetase (LysS), tryptophan-tRNA synthetase (TrpS), and GltX, while HipA_1_ phosphorylates TrpS and GltX (57). In both cases, the stringent response regulator SpoT (Rel) is required for *hipBA*_1_- and *hipBA*_2_-mediated persistence. A recent report confirmed that HipA_2_ only phosphorylates and inhibits TrpS, whereas LysS and GltX were not found to be phosphorylated (58). Nevertheless, both studies agreed that HipA_2_ induces the stringent response and persistence in parallel (57, 58).

We recently described a new family of minimal bacterial kinases, HipT, members of which exhibit sequence similarity with the C-terminal part of HipA but are encoded by three-gene operons (Fig. 1B) (59). HipT of E. coli O127 is functionally similar to HipA and phosphorylates tryptophanyl-tRNA synthetase (TrpS/TrpRS) at two conserved serine residues, inactivating the enzyme. Likewise, ectopic production of *hipT* inhibits cell growth and translation and, consistently, stimulates production of (p)ppGpp (59). The gene immediately upstream of *hipT* encodes a small protein, HipS, that exhibits sequence similarity to the N-terminal part of the larger HipA kinase (Fig. 1B). Surprisingly, HipS neutralizes HipT *in vivo*, and therefore appears to function as the antitoxin of the *hipBST* module. The third component, HipB, encoded by the first gene of the *hipBST* operon (Fig. 1B), contains an HTH domain and is homologous to HipB of HipBA but does not counteract HipT kinase activity directly. Rather, this protein functionally appears to augment the ability of HipS to neutralize HipT (59). The structural and mechanistic details that set the bicistronic and tricistronic Hip kinase systems apart have not yet been elucidated. Finally, E. coli K-12 carries genes that encode the HipA homologue YjjJ in a monocistronic operon, thus lacking an adjacent antitoxin gene, which also inhibits cell growth upon induction (Fig. 1C) (60). Interestingly, YjjJ contains a HTH domain at its N terminus that may compensate for the lack of a DNA-binding antitoxin; however, how YjjJ kinase activity is controlled remains unknown.

The discovery of the *hipBST* tricistronic operons (59) as well as the observed diversity among Hip toxin homologues in various bacterial species inspired us to investigate the overall phylogeny of HipA-homologous proteins and their gene families in prokaryotic microorganisms. This led to the discovery of seven novel Hip kinase families, potential antitoxins with novel features such as HIRAN (HIP116 and RAD5 N-terminal) domains with predicted specificity for single-stranded or double-stranded DNA ends and a novel putative two-domain antitoxin family consisting of a HipS-like domain coupled to a HIRAN domain. We also find evidence that HipA-homologous kinases are present in *Archaea*. Together, these results delineate the structural and functional diversity of the family of HipA kinases and suggest directions for future experimental research.

## RESULTS AND DISCUSSION

### HipA-homologous kinases form a strongly supported, bifurcated phylogenetic tree.

**FIG 2:**
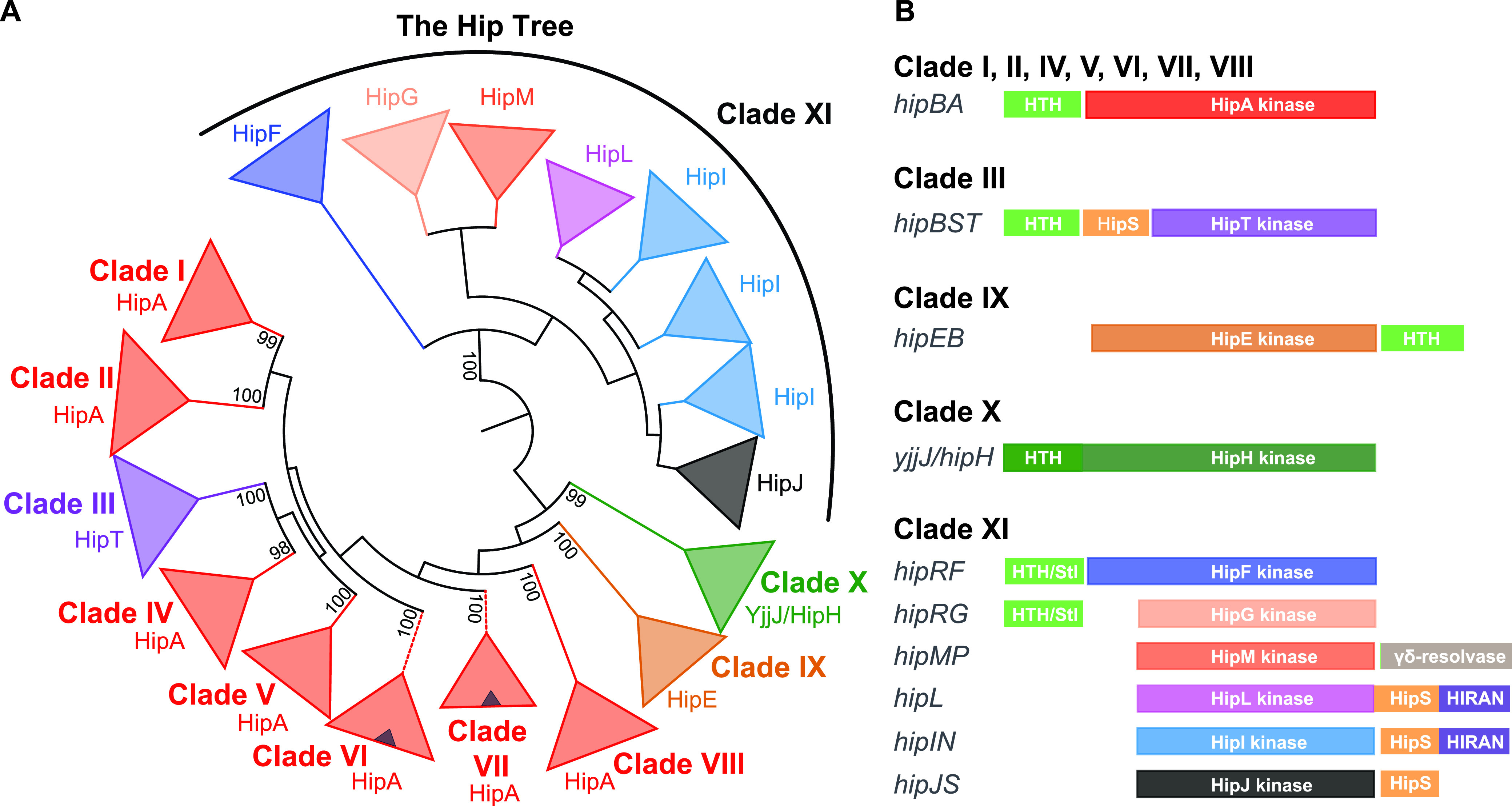
Phylogeny and genetic contexts of HipA-homologous kinases. (A) Simplified phylogenetic tree covering 1,239 HipA-homologous kinases (the “Hip Tree”) (see Fig. S2 in the supplemental material for details). The Hip Tree was divided into 11 main clades I to XI. The coloring of the Hip Tree reflects the genetic contexts that encode the kinases such that each color corresponds to a distinct kinase family encoded by a distinct type of TA module. All main clades are monophyletic except clade XI that consists of six different kinase families. Small blue triangles within the red triangles of main clades VI and VII symbolize subclades of kinases encoded by TA modules with a reversed gene order relative to *hipBA*—that is—with the gene order *hipAB*. The Hip Tree was visualized by iTOL (82). (B) Genetic organizations of the TA modules encoding the 1,239 HipA-homologous kinases. The various types of genetic organization were obtained by manual inspection of the genes upstream and downstream of the kinase genes listed in Table S1 in the supplemental material. The coloring of the Hip kinases in panel B follows the coloring of the clades in panel A. Putative antitoxins with helix-turn-helix (HTH) domains are colored light green. Stl/HTH, putative antitoxins containing HTH domain and a domain with structural similarity to the “polyamorous” repressor Stl encoded by Staphylococcus aureus; HipS, HipS-like; HIRAN, HIP116 Rad5p N-terminal domain.

The phylogenetic analysis was initiated using HipA and YjjJ of E. coli K-12 and HipT of E. coli O127 as seed sequences using BLASTP and HMMSEARCH (see Materials and Methods for details). While this revealed a vast number of HipA-homologous kinases within the bacterial domain and a few in the archaeal domain, searches in the *Eukarya* domains did not disclose significant homologues (E > 10^−5^). To analyze the vast number of high-score homologues (E > 10^−10^) systematically, we repeated the search using individual bacterial and archaeal phyla as search spaces (see Fig. S1 in the supplemental material) and retrieved ∼1,800 high-scoring Hip homologues. Tenacious curation, including manual inspection of putative neighboring antitoxin genes of each individual kinase gene, removal of incomplete genes, and exclusion of closely related kinases (<5% sequence difference) reduced the number of included kinases to a final set of 1,239 sequences. The majority of these are from the phylum *Proteobacteria* (70%) while they are also frequently observed among *Actinobacteria* (13%), *Firmicutes* (5%), and *Spirochaetes* (2%) (see Table S1 in the supplemental material). Using these sequences, we generated a phylogenetic tree, called the “Hip Tree” (Fig. 2A). Fully annotated and bootstrapped versions of the Hip Tree are shown in Fig. S2A and S2B, while a full breakdown of the phylogeny of the kinases is given in Table S2A. The Hip Tree consists of 11 main clades (clades I to XI) all supported by high statistical confidence levels (Table 1). The tree is bifurcated with one branch containing main clades I through X, while the other branch consists of the diverse main clade XI (Fig. 2A). In Fig. 2A, the colors of the clades (shown as triangles) reflect kinases encoded by TA modules with identical genetic organization, as explained in the next section. In other words, each differently colored triangle in Fig. 2A reflects HipA-homologous kinases encoded by TA modules with a different genetic organization.

**TABLE 1:** Overview of the clades of the Hip Tree consisting of 1,239 kinases^*a*^

Clade	No. of kinase genes	TA gene organization^*b*^	Experimentally analyzed TA modules	Cellular target(s) of toxin	GenBank ID^*c*^	Reference(s)
I	712	*hipBA*	*hipBA* of E. coli K-12	GltX	NP_416024.1 (440)	47, 51, 52, 57, 58
			*hipBA*_1_ of C. crescentus	Unknown or GltX and LysS	ACL93947.1 (423)	
			*hipBA*_2_ of C. crescentus	LysS or GltX, LysS and TrpS^*e*^	ACL96286.1 (444)	
			*hipBA*_3_ *of* C. crescentus	Unknown^*e*^	WP_010920611.1 (435)^*d*^	
			*hipBA* of S. oneidensis MR-1	Unknown	AAN53784.1 (433)	
II	73	*hipBA*	None			
III	48	*hipBA*	*hipBST* of E. coli O127	TrpS	WP_015879003.1 (342)	59
			H. influenzae KW20	TrpS	NP_438824.1 (343)	
			T. auensis DSM9187	TrpS	CAS11333.1 (335)	
IV	14	*hipBA*	None			
V	9	*hipBA*	None			
VI	36	*hipBA*	None			
VII	132	*hipAB* and *hipAB*	None			
VIII	21	*hipBA*	None			
IX	12	*hipEB*	None			
X	101	*hipH/yjjJ*	*hipH/yjjJ*		P39410.1 (443)	60
XI	81	*hipRF*, *hipRG*, *hipMP*, *hipL*, *hipIN*, and *hipJS*	None			

a Data compiled from Table S1 in the supplemental material.

b Gene organization refers to information retrieved from Table S1 and visualized schematically in Fig. 2B.

c GenBank identifiers (IDs) or accession numbers of the kinases are from Table S1 and their position in amino acids in the Hip Tree visualized in Fig. S2A and S2B in the supplemental material.

d *hipBA*_3_ of C. crescentus previously had the accession number NP_421566.1 and was used in Table S1.

e The substrate of HipA_1_ is either unknown (58) or GltX plus LysS (57), while the substrate of HipA_2_ is either TrpS (58) or GltX, LysS plus TrpS (57).

10.1128/mBio.01058-21.1 FIG S1 Hip kinases divided on the Tree of Life. Overview of prokaryotic phyla used as search spaces in BLASTP and HMMSEARCH procedures. HipA and HipH/YjjJ of E. coli K-12 and HipT of E. coli O127 were used as queries in BLASTP and HMMSEARCH searches using individual prokaryotic phyla as search spaces. The colored dots reveal the frequencies of Hip homologues of the individual phyla included in the analysis and are indicated in the inset. Kinase frequencies of individual phyla were collapsed to maintain clarity; the exact numbers are given in Table S2A. Download FIG S1, PDF file, 2.5 MB. Copyright © 2021 Gerdes et al. 2021 Gerdes et al. https://creativecommons.org/licenses/by/4.0/ This content is distributed under the terms of the Creative Commons Attribution 4.0 International license.

10.1128/mBio.01058-21.2 FIG S2 Phylogenetic tree of 1,239 HipA-homologous kinases. (A) The Hip Tree of Hip kinases annotated with phylogenetic origin. Roman numerals I to XI denote the 11 deep-branching main clades colored as in Fig. 2. Kinase labels reveal phylum, gene size (in codons), and GenBank identifier (from Table S1). Stippled branches in main clades V and VII indicate TA operons with a reversed gene order relative to *hipBA*. The positions of HipA of E. coli, HipA_1_, HipA_2_, and HipA_3_ of C. crescentus, HipA of S. oneidensis MR-1, HipT of E. coli O127, and YjjJ (HipH) of E. coli K-12 that have been investigated experimentally are indicated. The outer ring shows the phylogenetic origin of the individual kinases in color: *Proteobacteria* (blue), *Actinobacteria* (green), *Firmicutes* (red), *Planctomycetes*, *Verrucomicrobia*, and *Chlamydiae* (PVC) group (orange), *Archaea* (*Crenarchaeota*, *Euryarchaeota*, and “*Candidatus* Woesearchaeota”), *Spirochaetes* (light green), *Bacteroidetes* (magenta), Cyanobacteria (light cyan), *Deferribacteraceae* (brown), *Acidobacteria* (black), *Fibrobacter* (light gray), *Aquificae* and *Thermotogae* (dark gray), all other phyla (*Nitrospirae*, *Tenericutes*, *Fusobacteria*, *Chlorobi*, *Ignavibacteriae*, and *Chrysiogenales* (dark green). Clade I consists of 712 kinases all encoded by *hipBA* modules and encompasses HipA of E. coli K-12, HipA_1_ and HipA_2_ of C. crescentus, and HipA of S. oneidensis (Table 1). Clade II consists of 73 kinases, also encoded by *hipBA* loci but is distinct from clade I by its deep branching and the high bootstrap value (100%) that separates the two clades. Clade II is dominated by kinases from *Actinobacteria*. Clade III consists of all 48 HipT kinases encoded by the tricistronic *hipBST* modules. Clade IV consists of 14 kinases, all encoded by *hipBA* modules. Clade IV kinases are all from *Proteobacteria*. Clade V consists of nine kinases, all encoded by *hipBA* modules. Most of these kinases are from *Proteobacteria*. Clade VI consists of 36 kinases, 31 of which are encoded by canonical *hipBA* operons, while 5 are encoded by operons with a reversed gene order (i.e., *hipBA*). Clade VII consists of 136 kinases encoded by 133 *hipBA* operons, while 3 of the kinases are encoded by operons with a reversed gene order (i.e., *hipAB*). Clade VII kinases are encoded by a heterogenous set of organisms encompassing *Proteobacteria*, the PVC group, *Acidobacteria*, *Spirochaetes*, *Tenericutes*, and *Archaea*. Clade VIII consists of 21 kinases, all encoded by *hipBA* modules. Most of clade VIII kinases are from *Proteobacteria*, while a few are from *Nitrospirae* and *Archaea*. Clade IX consists of 12 HipE kinases all encoded by operons *hipEB* operons with a reversed gene order relative to *hipBA* (Fig. 2B). Clade X consists of 101 YjjJ/HipH kinases, all encoded by monocistronic operons. The majority of the YjjJ/HipH kinases are from *Proteobacteria*, while a few are from the PVC group or *Chrysiogenales*. Clade XI, the most complex clade, consists of 81 kinases encoded by six different genetic contexts and contains six novel kinase families (Fig. 2B). (B) Bootstrapped version of the Hip Tree shown in panel A. The Hip Tree is based on a sequence alignment of the 1,239 kinases listed in Table S1 and was generated by Clustal Omega (89), bootstrapped by IQ-TREE (84) via a module provided by CIPRES (http://www.phylo.org/) in Genious Prime and visualized by iTOL (82). The best-fit model chosen for phylogenetic tree reconstruction was WAG+F+R10 according to the Baysian information criterion (BIC). Download FIG S2, PDF file, 2.2 MB. Copyright © 2021 Gerdes et al. 2021 Gerdes et al. https://creativecommons.org/licenses/by/4.0/ This content is distributed under the terms of the Creative Commons Attribution 4.0 International license.

### HipA-homologous kinases are encoded by 10 different genetic organizations.

By careful investigation of the sequence data set in Table S1, we found that a consistent and biological meaningful definition of “Hip kinase family” can be based on the genetic context encoding the kinases. Using this classification, we identified a total of 10 Hip kinase families encoded by TA modules with 10 different genetic organizations (Fig. 2B). The frequencies of the TA modules with different genetic organizations are given in Table S2B.

Using this classification, all HipA kinases encoded by *hipBA* modules with an upstream HipB HTH antitoxin gene cluster together in clades I, II, and IV to VIII. Experimentally characterized *hipBA* modules from E. coli K-12 and C. crescentus are all in clade I (Fig. S2A). Interestingly, clades VI and VII each contain a subclade that have a reversed gene order (i.e., *hipAB*), indicating that gene reversion occurred independently several times during evolution (shown as blue triangles within red triangles in Fig. 2A and as branches with dashed lines in Fig. S2A). Similarly, clade IX consists entirely of kinases encoded by operons with a reversed gene order relative to *hipBA*, and the deep branching of this clade warrants the definition of a new kinase family that we call HipE (Fig. 2A). HipE kinases are encoded by *hipEB* operons that also encode putative HipB antitoxins with HTH domains (Fig. 2B). Clade III consists solely of HipT kinases encoded by *hipBST* operons, including the characterized locus from E. coli O127, while clade X consists entirely of YjjJ-like kinases encoded by monocistronic operons. Interestingly, clade XI is highly diverse and contains no less than six kinase families (Fig. 2A) encoded by different genetic contexts (Fig. 2B). To standardize the nomenclature, all genes encoding putative antitoxins with HTH domains have been named HipB except for HipB of *hipBST* and HipR of *hipRF* and *hipRG*. In all cases investigated, HipB functions as both an antitoxin and an autoregulator of transcription with the exception of the HipB protein encoded by the *hipBST* operons, which autoregulates transcription but does not function as an antitoxin as mentioned above (20, 59). As described later, HipR-encoded *hipRF* and *hipRG* operons also contain a HTH domain but exhibits structural similarity with the Stl repressor.

### Conserved HipA kinases appear to contain functionally significant differences.

**FIG 3:**
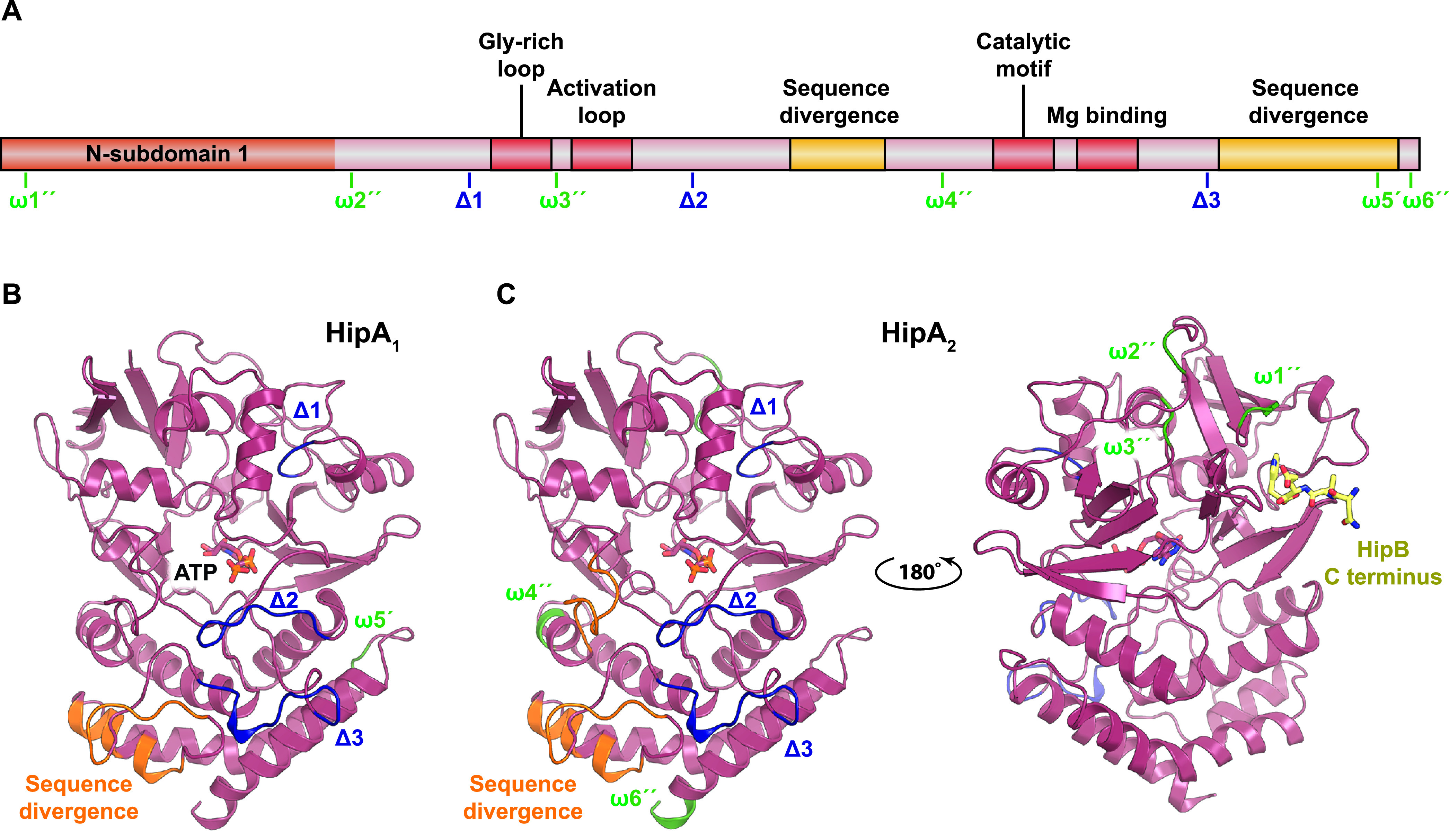
Structural mapping of insertions and deletions observed in characterized HipA kinases. (A) Schematic overview of HipA showing the conserved regions (the Gly-rich loop, activation loop, catalytic motif, and Mg-binding motif) in red as well as insertions (ω [green]) and deletions (Δ [blue]) in C. crescentus HipA_1_ and HipA_2_ relative to HipA from E. coli K-12 based the sequence alignment shown in Fig. S3. An insertion in HipA_1_ relative to HipA is denoted ω5′, while insertions in HipA_2_ relative to HipA are marked ω1″, ω2″, ω3″, ω4″, and ω6″. Deletions in HipA_1_ and HipA_2_ relative to HipA are marked Δ1, Δ2, and Δ3. Regions of high sequence divergence are shown in yellow. (B) Mapping of the insertions and deletions in HipA_1_ onto the structure of HipA of E. coli K-12 (PDB accession no. 3FBR) using the same nomenclature and color scheme as in panel A (45). ATP is shown with colored sticks. (C) Mapping of the insertions and deletions in HipA_2_ onto the structure of HipA. Note that the insertions ω1″ and ω3″ in HipA_2_ are located close to the region that in HipA interacts with the C terminus of HipB (yellow sticks) and could perhaps affect antitoxin activity.

Clade I contains the “classical” bacterial HipA kinases with known cellular targets, HipA of E. coli K-12, HipA_1_ and HipA_2_ of C. crescentus, together with their close homologues. An alignment of representative sequences of subclades containing HipA, HipA_1_, and HipA_2_ kinases reveals, as expected, the four canonical core kinase motifs: the Gly-rich loop, the activation loop, the catalytic motif, and the Mg^2+^-binding motif (Fig. S3). The alignment also reveals a number of insertions (ω1 to ω6) and deletions (Δ1 to Δ3), also called “indels” in the two C. crescentus HipA (HipA*_Ccr_*) subclades relative to the E. coli K-12 HipA (HipA_E. coli__K-12_) subclade (Fig. S3). Figure 3A shows a schematic overview of HipA indicating the positions of the indels relative to the core kinase motifs in the primary sequences, while Fig. 3B and C show a mapping of the HipA_1_- and HipA_2_-specific indels as well as regions of high sequence divergence onto the structure of HipA of E. coli K-12. Interestingly, despite being distant in the primary sequence, the two largest deletions in E. coli HipA (Δ2 and Δ3) are adjacent in the tertiary structure (Fig. 3B and C) and close to the γ-phosphate of ATP. Additionally, both C. crescentus HipA kinases share a C-terminal region of high sequence divergence that maps to solvent-exposed residues of a surface helix (Fig. 3A), while HipA_2_ has an additional unique region of divergence (Fig. 3C). Studies of eukaryotic cyclin-dependent kinases have shown that the homologous region where Δ2, Δ3, and both regions of divergence are situated is involved in target binding (61), raising the possibility that the differences observed relate to differences in target specificity. Finally, we note that HipA_2_ has several regions (ω1″, ω2″, and ω3″) not present in HipA (Fig. 3A and Fig. S3) concentrated in the region that interacts with the very C terminus of HipB in the E. coli HipBA structure (Fig. 3C), thus potentially affecting the mechanism by which the antitoxin interacts with and inhibits the cognate kinase.

### All HipT kinases lack the canonical N-terminal subdomain.

Main clade III consists exclusively of HipT kinases (Fig. 2). As shown before, the sequences of the HipT kinases of E. coli O127, Haemophilus influenzae, and Tolumonas auensis align colinearly with the C-terminal part of E. coli K-12 HipA (Fig. S4A) but lack the canonical ∼100-amino-acid (aa) N-subdomain-1 that serves as a lid on top of the core kinase domain in HipA (59). Nevertheless, the four conserved Hip kinase motifs (Gly-rich loop, activation loop, catalytic motif, and Mg^2+^-binding motif) are conserved in the HipTs, as well as a serine adjacent to the Gly-rich loop that is subject to autophosphorylation in both HipT and HipA (46, 59). We showed previously that HipS, which is encoded immediately upstream of HipT, functions as the antitoxin of *hipBST* modules and that this protein exhibits sequence similarity to the N-terminal domain of HipA (59) (Fig. S4A and S4B). In other words, the “missing” N-subdomain-1 of HipT appears to be encoded immediately upstream and thus in a similar genomic location relative to the core kinase domain as in HipA, which suggests that the gene has been split (or merged) at some point during evolution. However, the functional and structural implications of this difference with respect to kinase activation and regulation are not yet understood.

10.1128/mBio.01058-21.3 FIG S3 Alignment of HipA kinases that have different cellular targets. Sequences of subclades containing HipA of E. coli K-12, HipA_1_ and HipA_2_ of C. crescentus NA1000 were aligned. The subclades were selected such that they are separated by high bootstrap values (Fig. S2). Deletions (Δ) and insertions (ω) relative to the HipA_E. coli__K-12_ subclade are indicated as well as the four conserved kinase motifs (Gly-rich loop, activation loop, catalytic motif, and Mg^2+^-binding motif). Clade-specific insertions in HipA_1_ and HipA_2_ are indicated with red boxes. Download FIG S3, JPG file, 2.6 MB. Copyright © 2021 Gerdes et al. 2021 Gerdes et al. https://creativecommons.org/licenses/by/4.0/ This content is distributed under the terms of the Creative Commons Attribution 4.0 International license.

10.1128/mBio.01058-21.4 FIG S4 HipT and HipS exhibit sequence similarity with the C- and N-terminal ends of HipA, respectively. (A) Subclade HipA_E. coli__K-12_ of main clade I aligned with subclade HipT_E. coli__O127_ of main clade III (Fig. S2). The four conserved canonical Hip kinase motifs are indicated as well as a conserved, autophosphorylated serine residue. Conserved deletions (Δ) and insertions (ω) in the HipT kinases relative to the HipA kinases are also indicated. (B) Subclade HipA_E. coli__K-12_ aligned with subclade HipS_E. coli__O127_ antitoxins. Download FIG S4, PDF file, 2.8 MB. Copyright © 2021 Gerdes et al. 2021 Gerdes et al. https://creativecommons.org/licenses/by/4.0/ This content is distributed under the terms of the Creative Commons Attribution 4.0 International license.

### YjjJ kinases contain an N-terminal HTH domain.

**FIG 4:**
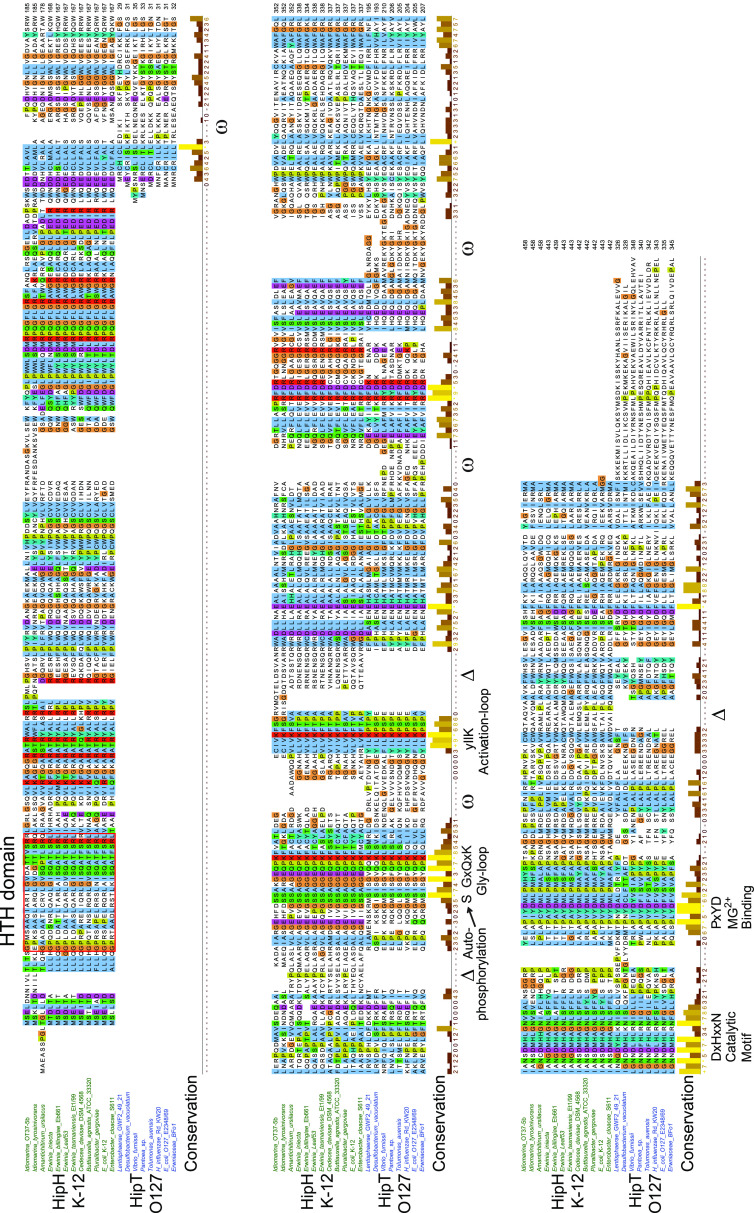
Alignment of HipH and HipT kinases. Sequence alignment of subclades containing HipT of E. coli O127 and HipH of E. coli K-12. Deletions (Δ) and insertions (ω) relative to the HipH*_E coli_*_K-12_ subclade are indicated, as well as the four conserved kinase motifs (Gly-rich loop, activation loop, catalytic motif, and Mg^2+^-binding motif). HTH domains in the N terminus of HipH kinases are boxed in red.

YjjJ of E. coli K-12 is a HipA homologue encoded by a monocistronic operon and thus “lacking” a closely linked antitoxin or DNA-binding HipB-like gene. Instead, YjjJ has a ∼100-aa N-terminal extension containing a helix-turn-helix (HTH) domain (residues 15 to 34) that potentially could function as a *cis*-acting antitoxin, a property previously found with other type II modules (62). To maintain a uniform nomenclature, we propose here to rename YjjJ kinases HipH (H for HTH domain) as these constitute the monophyletic clade X in the Hip Tree (Fig. 2A). To avoid alignment of nonhomologous domains, we chose to align subclade HipH_E. coli__K-12_ with HipT_E. coli__O127_ because HipT lacks the ∼100-aa N-terminal domain present in HipA but has all the canonical kinase domains. As seen from Fig. 4, HipH and HipT kinases align colinearly with respect to the four conserved kinase motifs, while the N terminus of HipH forms a separate HTH domain. Moreover, HipH kinases contain two conserved serine residues near the Gly-rich loop, raising the possibility that they are regulated through autophosphorylation in a way similar to HipT (59).

### Main clade XI consists of kinases belonging to six different families.

The kinases in main clade XI of the Hip Tree are encoded by six different genetic contexts and define six novel Hip kinase families (Fig. 2A and B). All six subclades are separated by high bootstrap values, supporting that their separation into novel kinase families is phylogenetically justified (Fig. S5). The six families encompass four families of short kinases (HipG, HipM, HipI, and HipJ), one longer variant (HipF) similar in size to HipA and a “long” kinase family (HipL) (Fig. 2B). In the following, we describe these six new types of kinase-encoding TA modules.

### The HipG, HipM, HipI, and HipJ families contain the core kinase domain.

The HipG, HipI, HipJ, and HipM kinases are all phylogenetically closely related and are, on average, even smaller than the HipT kinases (279 to 321 aa versus 291 to 346 aa, respectively) (Table S1 and Fig. S5). A sequence alignment of representatives of these families confirms that they contain the four conserved core Hip sequence motifs are thus likely active kinases (Fig. S6). However, like HipT, these kinases lack the N-subdomain-1 present in HipA, HipE, and HipF and consequently, the Gly-rich loop is located close to the N termini (Fig. S6). The kinases in the HipG group do not have a conserved serine or threonine adjacent to the Gly-rich loop, suggesting they are probably not regulated by autophosphorylation in a way similar to HipA. In contrast, members of the other families (HipM, HipI, and HipJ) mostly have either Ser or Thr near the Gly-rich loop, suggesting on the other hand that they may be regulated in this way. We note that HipI kinases constitute four separate subclades of the phylogenetic reconstruction of main clade XI (Fig. 2A). Three of the subclades consists of kinases from *Actinobacteria* and *Firmicutes* while the fourth consists of a mix of kinases from *Proteobacteria* and Cyanobacteria (Fig. S5).

### HipF and HipG kinases have putative antitoxins related to polyamorous repressor Stl.

HipF and HipG kinases are encoded in operons with putative antitoxins that we call HipR (Fig. 2B). Interestingly, Phyre2 (63) predicts HipR to be structurally related to the two-domain transcriptional repressor Stl encoded by Staphylococcus aureus superantigen-carrying pathogenicity islands (*Sa*PI). Stl has an HTH domain and maintains integration of *Sa*PI elements in the bacterial chromosome by transcriptionally repressing genes essential for element excision and replication (64). *Sa*PI excision and replication are induced by invading phages via specific interaction between Stl and nonessential phage proteins (65–69). These observations allow us to hypothesize that phage proteins could potentially activate HipF and/or HipG kinases during infection via interaction with HipR. Under this model, activation of the kinases induces abortive phage infection by inhibition of translation via phosphorylation of an essential component of the protein synthesis apparatus, thus eliciting phage resistance. This possibility can now be tested experimentally.

### Putative HipN antitoxins consist of a HipS-like and a HIRAN domain.

The genes encoding HipM, HipI, and HipJ kinases all have downstream genes coding for putative antitoxins (Fig. 2B). The putative antitoxin HipN encoded by *hipIN* is a two-domain protein consisting of an N-terminal HipS-like domain (Fig. S7A) and a C-terminal HIRAN domain (Fig. S8A). The HipS-like domain of HipN may function to neutralize its cognate HipI kinase as is the case of HipS encoded by *hipBST* (59). A possible function of the HIRAN domain of HipN is discussed below.

### A second family of putative HipS antitoxins.

*hipJS* modules encode a HipJ kinase and a putative antitoxin HipS that exhibits similarity to HipS of *hipBST* (Fig. S7A). Thus, similarly to HipT (59), HipJ may be neutralized by its cognate HipS. As noted above, HipS and HipS-like domains exhibit sequence similarity with the ∼100-aa N-subdomain-1 of HipA, and this domain was therefore included in the alignment of the HipS antitoxins and HipS-like domains (Fig. S7A). The phylogenetic tree based on the HipS and HipS-like sequences is bifurcated, with HipS encoded by *hipBST* and *hipJS* branch together with N-subdomain-1 of HipA, while the HipS-like sequences encoded by *hipL* and *hipN* generate a distinct second branch (Fig. S7B). HipJ kinases are closely related to HipI kinases (Fig. S5), and it is tempting to speculate that *hipJS* modules evolved from *hipIN* modules by deletion of the HIRAN domain of HipN antitoxins (Fig. 2B).

### HipL kinases contain both HipS-like and HIRAN domains.

HipL kinases, encoded by monocistronic operons, form a single subclade of main clade XI that is further divided into two subclades (Fig. S5). The two subclades consist of HipL kinases from Gram-positive and Gram-negative bacteria. Their sequences are clearly distinct, with many subclade-specific insertions and deletions in the kinase core domain (Fig. S8B). Alignment of HipL and HipA reveals that HipL maintains the four, conserved core kinase motifs and has a large C-terminal extension of ∼200 aa of which the ∼100 aa at the extreme C terminus are annotated at GenBank as a HIRAN domain (Fig. S8C). Like the small kinases, HipL kinases lack the N-subdomain-1 of ∼100 aa relative to the core kinase domain of HipA (Fig. S8C). Unexpectedly, however, the ∼100-aa domain located between the core kinase domain and the C-terminal HIRAN domain exhibits sequence similarity with HipS (Fig. S8D). The N-terminal sequences of HipA also align well with the HipS-like domain of HipL (Fig. S8E), consistent with the fact that HipS exhibits sequence similarity with the N-subdomain-1 of HipA (Fig. S4B). In summary, HipL kinases are three-domain proteins consisting of an N-terminal kinase core domain, a HipS-like domain, and a C-terminal HIRAN domain (Fig. 2B and Fig. S8C). We note the possibility that the HipS-like domain may be involved in regulating the kinase activity of HipL, reminiscent of how HipS regulates the kinase activity of HipT (59).

### The HIRAN domains of HipL and HipN may bind DNA.

**FIG 5:**
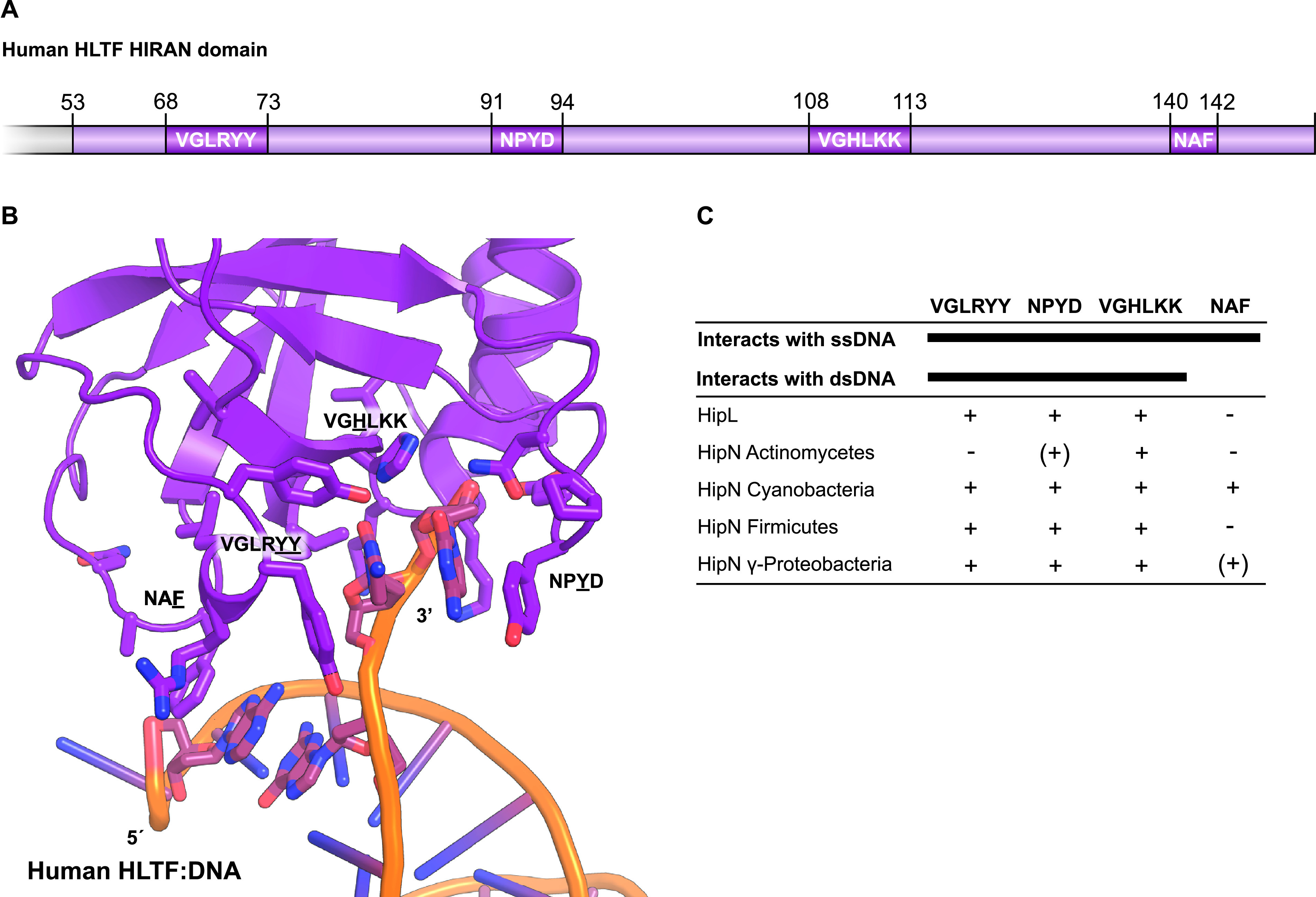
Comparative analysis of HIRAN domains. The HIRAN domains of HipL and HipN have sequence motifs required for DNA binding. (A) Schematic overview of the HIRAN domain found in human helicase-like transcription factor (HLTF) showing the relative positions and sequences involved in ssDNA and dsDNA binding (72). (B) Structure of the human HLTF HIRAN domain with the four regions necessary for DNA binding shown as sticks (PDB accession no. 4XZF) (72). (C) Overview of sequence motifs present (+) or absent (−) in homologues of HipL and HipN.

The presence of HIRAN domains in both HipL kinase and the putative antitoxins HipN is interesting, not least because HIRAN domains can bind DNA. Moreover, the HIRAN domains of HipL kinases and HipN antitoxins are in both cases joined with a HipS-like domain (Fig. 2B). HIRAN domains have previously been identified in eukaryotic multidomain DNA repair proteins (70). Experimentally analyzed HIRAN domains bind single-stranded or double-stranded DNA ends (71, 72). Structural studies of human helicase-like transcription factor (HLTF), a DNA helicase implicated in remodeling of replication forks, including fork regression and restart (73), revealed the residues required for DNA binding (72) (Fig. 5A and B). Due to a high sequence divergence, we decided to split the HIRAN domains of the HipN homologues based on their phylogenetic origin (Fig. 5C and Fig. S8A). Most of the HIRAN domains retained the majority of the sequence motifs that interact with DNA, with the exception of HipN from *Actinobacteria*. A study of the HIRAN domain of human HLTF showed that Phe142 (NAF) is required for binding to duplex DNA because it stacks with nucleobases of the other strand (72) (Fig. 5B). Importantly, most of the bacterial HIRAN domains lack a conserved Phe at this position (Fig. 5C), raising the possibility that the HIRAN domains in HipN and HipL interact with single-stranded DNA (ssDNA).

The function(s) of the HIRAN domains of HipL and HipN is, of course, unknown. One attractive possibility is that the HIRAN domains of HipL and HipN recognize DNA ends (ssDNA or double-stranded DNA [dsDNA] or both) of invading phages or phage DNA replicative intermediates that, in turn, leads to activation of the HipL and HipI kinases. Activation of the kinases could lead to phosphorylation and inhibition of an essential cellular component, thereby triggering abortive phage infection. Abortive phage infection is a common biological function of TA modules (74, 75), and the possibility that *hipL* and *hipIN* function to curb phage infection can now be tested experimentally.

### A putative antitoxin with a γδ-resolvase-like domain.

A final variation among the Hip kinases is found for the *hipMP* modules, which encode a HipM kinase that is short and closely related to HipG kinases (Fig. 2A) and a putative HipP antitoxin that exhibits similarity to γδ-resolvases (Fig. 2B). Bacterial γδ-resolvases are transposon-encoded enzymes that catalyze recombination within a complex nucleoprotein structure during site-specific DNA recombination and thus have the capability to bind DNA (76). Interestingly, γδ-resolvases are structurally related to 5′–3′ exonucleases active on RNA, which may also provide clues to the role of this domain in the context of Hip kinases (77). By analogy with other *hipBA* modules (28, 78), we therefore postulate that HipP functions as antitoxin in the *hipMP* systems and may bind DNA to autoregulate transcription as seen for many type II TA systems.

### Conclusion.

In addition to the three known kinase families HipA, HipT, and YjjJ/HipH, we discover here seven novel Hip kinase families encoded by different genetic contexts. Kinases of one family, HipE, are encoded by TA operons with a reversed gene order relative to *hipBA*, while kinases of families HipF and HipG have putative Stl-homologous antitoxins that may be regulated by proteins encoded by attacking phages. Kinases of one family, HipJ, are associated with HipS domain putative antitoxins that, similar to HipS of *hipBST*, may interact with and neutralize their cognate kinase toxins. Kinases of yet another family, HipI, are associated with putative antitoxins that consist of a HipS-like domain and a HIRAN DNA-binding domain, while HipJ kinases are associated with putative antitoxins exhibiting similarity with γδ-resolvases. Finally, HipL kinases, encoded by monocistronic operons, consist of an N-terminal core kinase domain, an internal HipS-like domain, and a C-terminal HIRAN DNA-binding domain. The latter two domains of HipL may function in regulating its kinase activity. Our analysis presented here builds a foundation for the future experimental analysis of HipA-homologous kinases.

## MATERIALS AND METHODS

### Data sampling.

As of December 2019, HipA and HipH/YjjJ of E. coli K-12 and HipT of E. coli O127 were used as seeds in BLASTP searches at https://blast.ncbi.nlm.nih.gov/, using the bacterial phyla shown in Fig. S1 as search spaces. HMMSEARCH at www.ebi.ac.uk (79) was used to expand poorly populated clades. In total, ≈1,800 Hip sequences were retrieved (E > 10^−10^) and curated manually such that every Hip kinase sequence retained satisfied the following criteria. (i) The Hip kinase gene should encode a full-length kinase with the four canonical kinase motifs (Gly-rich loop, activation loop, catalytic motif, and Mg^2+^ binding domain), as determined from a multiple-sequence alignment (MSA). (ii) Kept kinases should be encoded by a gene with an adjacent upstream or downstream putative antitoxin gene. (iii) In general, the adjacent, putative antitoxin gene should encode a DNA-binding protein (although this criterion is not satisfied by HipJ proteins). In Table S1 in the supplemental material, kept kinases are less than 95% identical to any other kept kinase. By this scrutiny, the initial gene set was reduced to 1,239 Hip gene modules (Table S1 and Fig. 2).

### Toxin-antitoxin module gene organization.

The gene organizations shown in Fig. 1 were deduced by manual inspection of genes neighboring the Hip kinase genes of Table S1.

### Sequence alignments and phylogenetic tree reconstruction.

Sequence alignments were generated by Clustal Omega (80) at www.ebi.ac.uk and imported into Jalview (81). Protein sequence alignments in Jalview 2.11.0 were exported as vector files (EPS or SVG formats) and imported into Adobe Illustrator CS6, annotated, and saved in PDF format for publication. Phylogenetic trees were visualized using iTOL (82). Reconstruction of phylogenetic trees was accomplished using IQ-TREE that uses the maximum likelihood approach and Ultrafast bootstrapping via the CIPRES module in Genious Prime (83–85). The kinase sequence alignments used in the reconstruction of the three phylogenetic trees that we present had the following characteristics. (i) The sequence alignment of the 1,239 kinases (Fig. S2) has 1,556 columns, 1,511 distinct patterns, 1,172 parsimony-informative sites, 172 singleton sites, and 211 constant sites. (ii) The sequence alignment of the 81 sequences of main clade XI (Fig. S5) has 785 columns, 761 distinct patterns, 650 parsimony-informative sites, 58 singleton sites, and 77 constant sites. (iii) The sequence alignment of the 112 HipS and HipS-like sequences (Fig. S7B) has 170 columns, 170 distinct patterns, 146 parsimony-informative sites, 16 singleton sites, and 8 constant sites. The alignment of the HipS and HipS-like sequences has fewer singleton and constant sites, thus explaining the low bootstrap values (Fig. S7B).

Structure similarity searches were done using Phyre2 (63) (http://www.sbg.bio.ic.ac.uk/phyre2/) and mapping of deletions and insertions on existing structures using PyMOL. HTH motifs were identified by two different algorithms, EMBOSS (86) and HELIX-TURN-HELIX MOTIF PREDICTION (87).

10.1128/mBio.01058-21.5 FIG S5 Phylogenetic tree of main clade XI. The clade consists of 81 kinases belonging to the six novel Hip families HipF, HipG, HipI, HipJ, HipM, and HipL. HipF and HipL kinases (encoded by *hipRF* and *hipL* loci) are located in single subclades, while HipG, HipI, HipJ, and HipM are located in four different subclades where the kinases are encoded by TA modules with different genetic organizations (*hipRG*, *hipIN*, *hipJS*, and *hipMP*). The genetic organizations are shown schematically in Fig. 2B. The HipG, HipI, HipJ, and HipM kinases have similar sizes and are phylogenetically related; however, the bootstrap values and the subclade-specific genetic organizations warrant their designation as separate kinase families. The best-fit model chosen for phylogenetic tree reconstruction was LG+F+R5 according to BIC. Download FIG S5, PDF file, 0.3 MB. Copyright © 2021 Gerdes et al. 2021 Gerdes et al. https://creativecommons.org/licenses/by/4.0/ This content is distributed under the terms of the Creative Commons Attribution 4.0 International license.

10.1128/mBio.01058-21.6 FIG S6 Alignment of the HipG, HipI, HipJ, and HipM kinases of clade XI. The four conserved Hip kinase motifs are indicated. The kinases of these four families are encoded by TA modules with four different genetic organizations (Fig. 2B) and have very short N-terminal domains. Download FIG S6, JPG file, 2.9 MB. Copyright © 2021 Gerdes et al. 2021 Gerdes et al. https://creativecommons.org/licenses/by/4.0/ This content is distributed under the terms of the Creative Commons Attribution 4.0 International license.

10.1128/mBio.01058-21.7 FIG S7 Sequence alignments and phylogeny of HipS and HipS-like domains. (A) Alignment of HipS encoded by *hipBST* modules with HipS-like domains of HipN encoded by *hipIN*, HipS encoded by *hipJS*, and HipS-like domain encoded by *hipL*. For comparison, the N terminus of HipA was included in the alignment. (B) Phylogenetic tree derived from the sequence alignment shown in panel A. The best-fit model chosen for reconstruction of the phylogenetic tree was LG+R5 according to BIC. Download FIG S7, JPG file, 2.7 MB. Copyright © 2021 Gerdes et al. 2021 Gerdes et al. https://creativecommons.org/licenses/by/4.0/ This content is distributed under the terms of the Creative Commons Attribution 4.0 International license.

10.1128/mBio.01058-21.8 FIG S8 Alignment of HIRAN domains and of HipL kinases. (A) The HIRAN domain of human HLTF aligned with the HIRAN domains of HipL kinases and putative HipN antitoxins. The sequence motifs of the HIRAN domain of HLTF involved in interaction with DNA are shown in boxes and are color coded (see also Fig. 5). The HipN sequences were divided into four separate alignments according to phylogeny in order to reveal the similarities with the sequence motifs of the HIRAN domain of HLTF. Color codes of the individual sequence motifs: black indicates conserved motifs that may have the capability to interact with DNA, red indicates that conserved motifs were not identified, and orange indicates some conservation. (B) Alignment of the HipL kinases of main clade XI. The four core kinase motifs are indicated. The alignment reveals two subclades consisting of kinases from Gram-positive and Gram-negative species, respectively (also apparent from Fig. S5). (C) Alignment of HipL kinases with subclade HipA_E. coli__K-12_. Deletions (Δ) and insertions (ω) in HipL relative to HipA, as well as the core kinase motifs are indicated. (D) Alignment of HipL kinases with HipS antitoxin sequences (subclade HipS_E. coli__O127_). As seen, the internal domain of HipL exhibits sequence similarity with HipS. (E) Alignment of HipL kinases with the N-terminal domains of HipA_E. coli__K-12_ kinase subclade. Download FIG S8, JPG file, 2.8 MB. Copyright © 2021 Gerdes et al. 2021 Gerdes et al. https://creativecommons.org/licenses/by/4.0/ This content is distributed under the terms of the Creative Commons Attribution 4.0 International license.

10.1128/mBio.01058-21.9 TABLE S1 Primary information on 1,239 HipA-homologous kinases. The columns A to P yield the following information. (A) GenBank ID of kinase toxins. (B) Kinase gene lengths in codons. (C) Organism. (D) Label yielding phylum, gene length in codons. and GenBank ID used the Hip Tree (Fig. S2). (E) Kinase sequences. (F) Distance between kinase toxin and its cognate antitoxin in nucleotides (a minus indicates gene overlap). (G) Genetic organization (GO) of each toxin-antitoxin module. (H) Antitoxin GenBank ID. (I) Antitoxin gene length in codons. (J) Label yielding antitoxin phylum, gene length, and GenBank ID. (K) Antitoxin sequence including HipBs encoded by *hipBST* loci (HipS antitoxin sequences have their own dedicated columns in the table). (L) Label yielding HipS phylum, gene length. and GenBank ID (used in HipS alignments of Fig. S4B, Fig. S7A, and Fig. S8D). (M) Distance between *hipS* and *hipT* in nucleotides. (N) HipS antitoxin sequences. (O) Domains in antitoxins. (P) Comments to individual entries in the table. GO, genetic organization. The full names of phyla are given in Table S2A. Download Table S1, TXT file, 0.7 MB. Copyright © 2021 Gerdes et al. 2021 Gerdes et al. https://creativecommons.org/licenses/by/4.0/ This content is distributed under the terms of the Creative Commons Attribution 4.0 International license.

10.1128/mBio.01058-21.10 Table S2 (A) The 1,239 Hip kinases divided by phyla. The numbers were extracted from the data in Table S1. (B) Frequencies of gene organizations of the 1,239 Hip Tree. Gene organizations of TA modules encoding Hip kinase toxins and their frequencies summarized from Table S1. Download Table S2, DOCX file, 0.02 MB. Copyright © 2021 Gerdes et al. 2021 Gerdes et al. https://creativecommons.org/licenses/by/4.0/ This content is distributed under the terms of the Creative Commons Attribution 4.0 International license.
