# Supplementary figures and images for: Phylogeny Reveals Novel HipA-Homologous Kinase Families and Toxin-Antitoxin Gene Organizations
Source: mBio. 2021 Jun 1;12(3):e01058-21. doi: 10.1128/mBio.01058-21 (PMC8262856; doi:10.1128/mBio.01058-21)

Figure S1

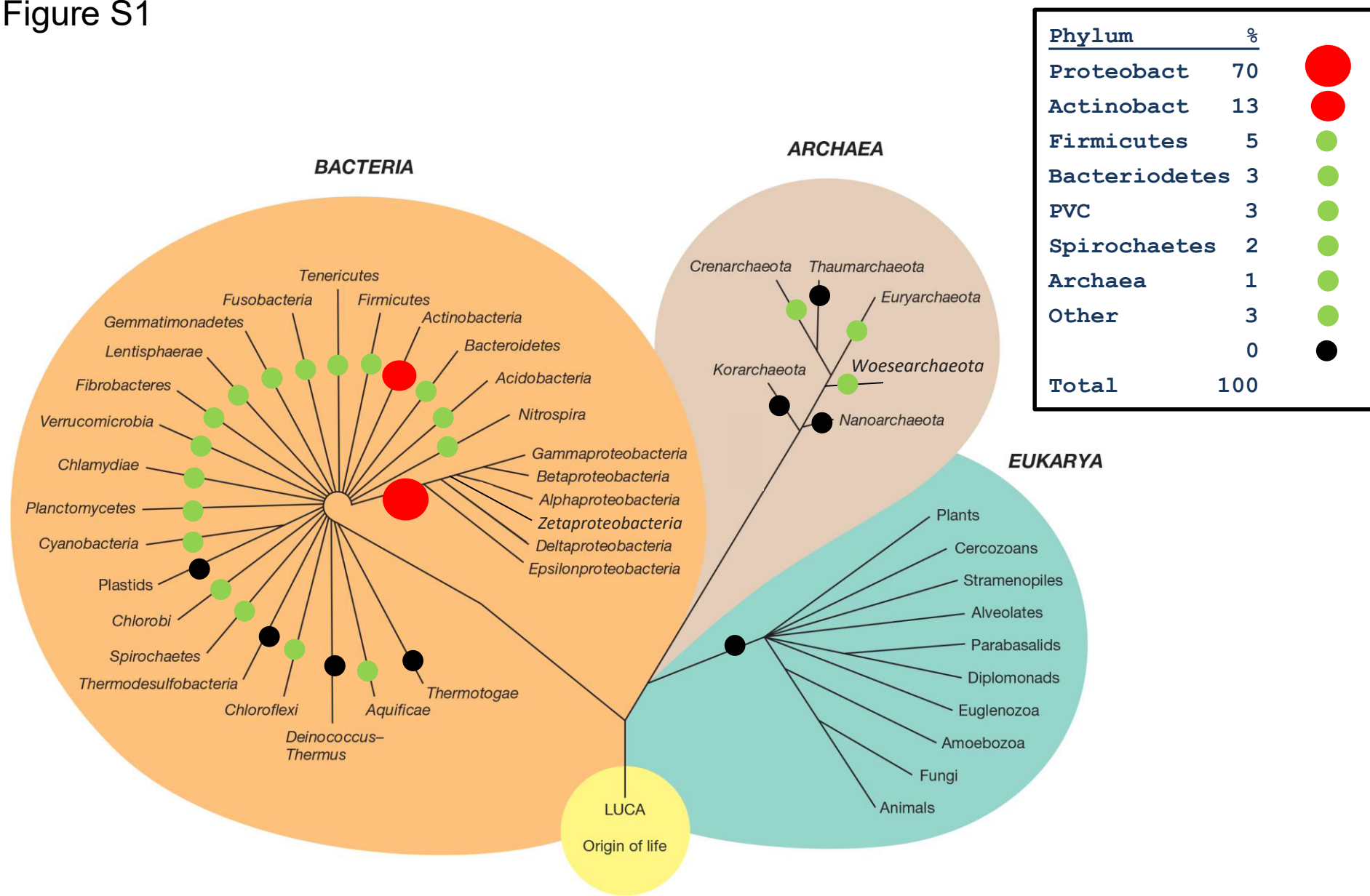

Supplement: FIG S1 [file mbio.01058-21-sf001.pdf]

Figure S2A

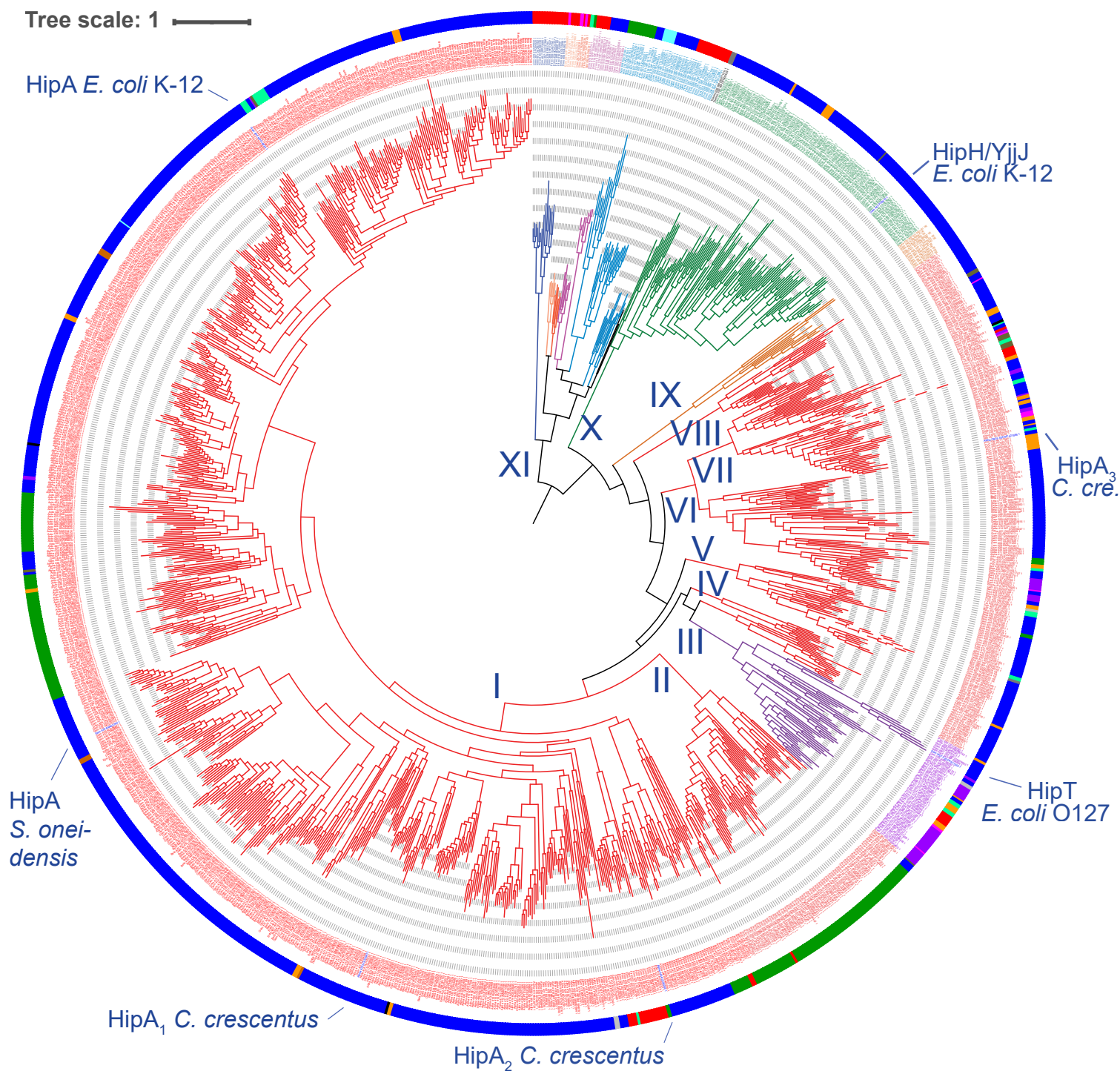

Figure S2B

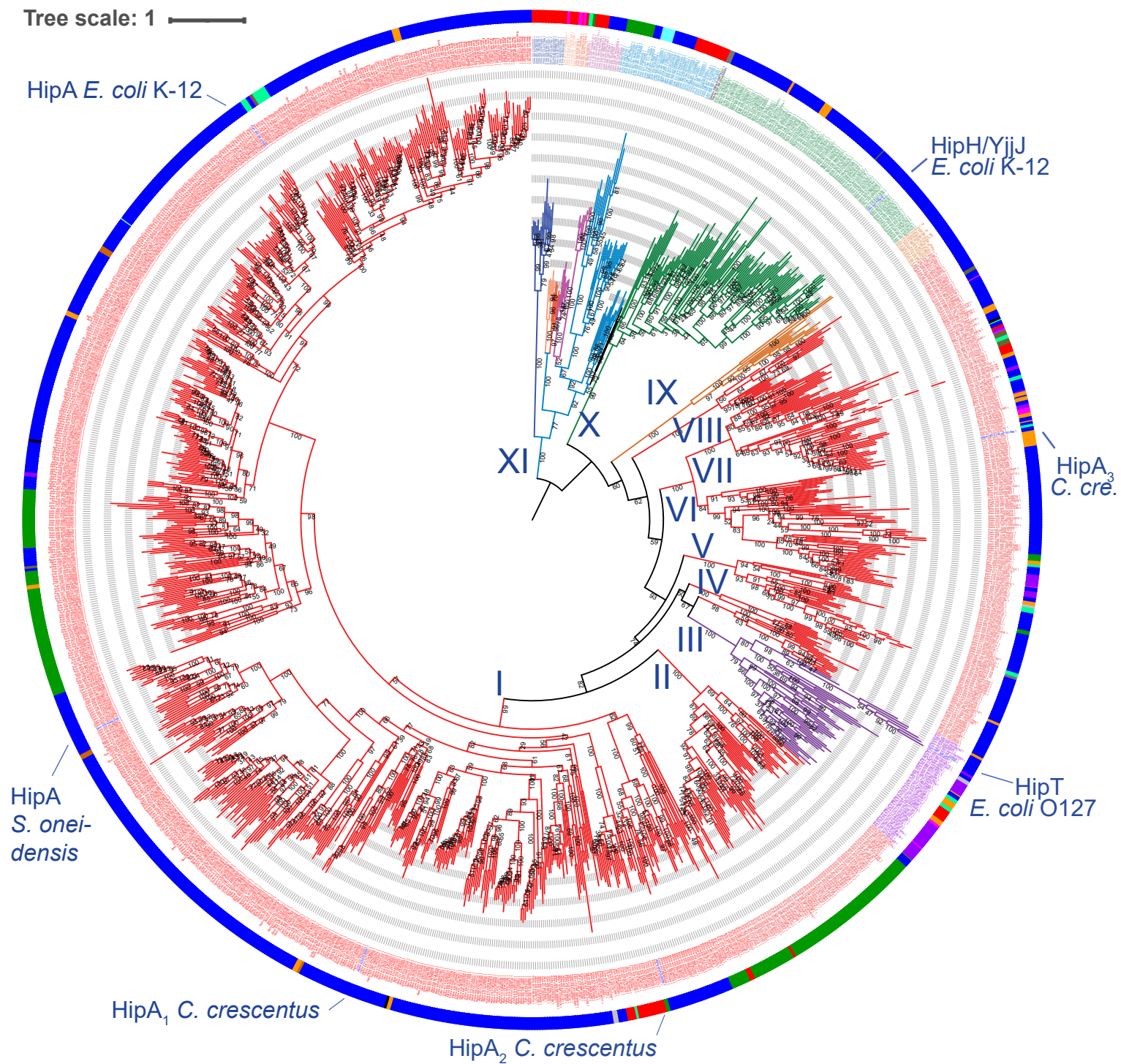

Supplement: FIG S2 [file mbio.01058-21-sf002.pdf]

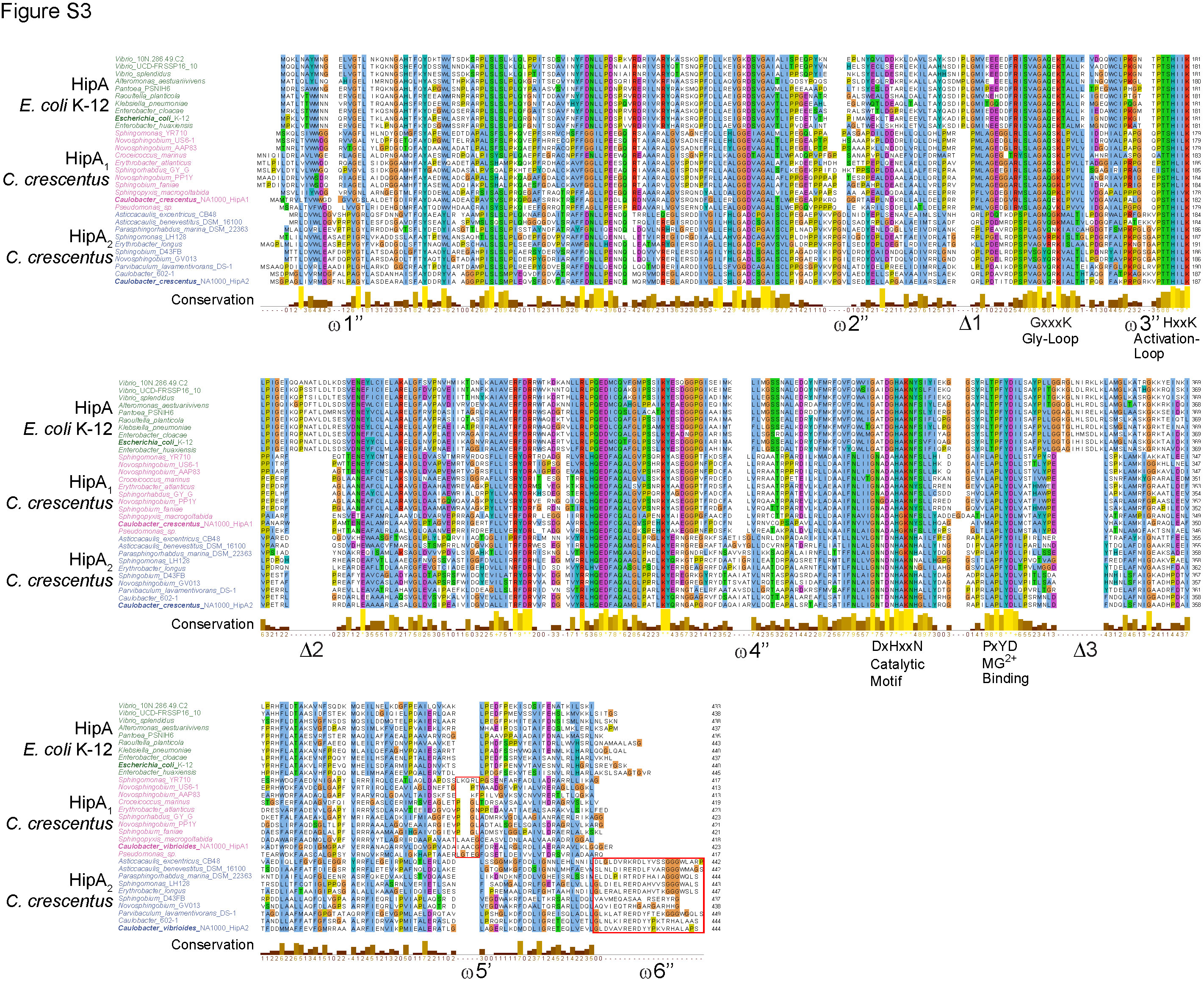

Supplement: FIG S3 [file mbio.01058-21-sf003.jpg]

Figure S5

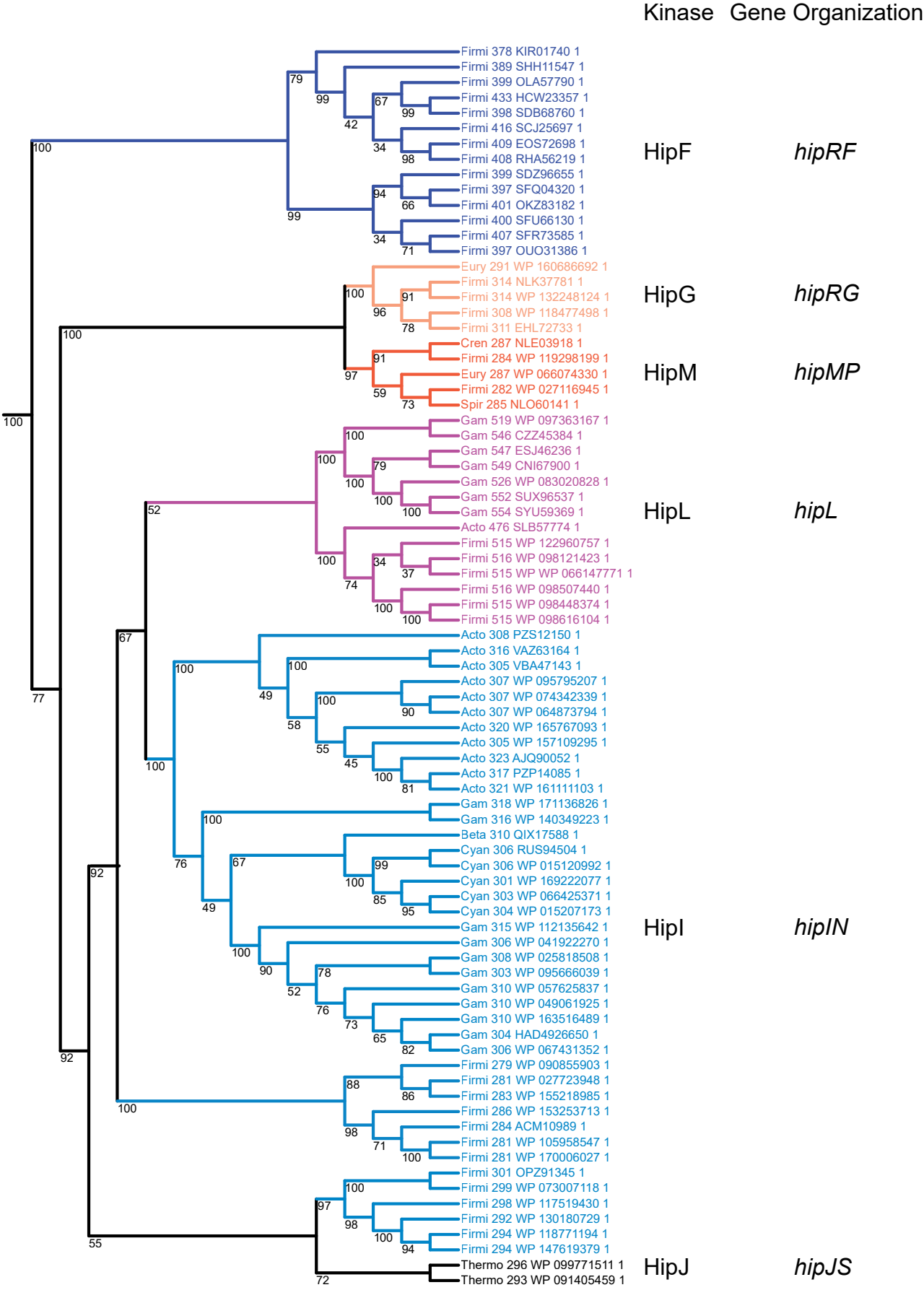

Supplement: FIG S5 [file mbio.01058-21-sf005.pdf]

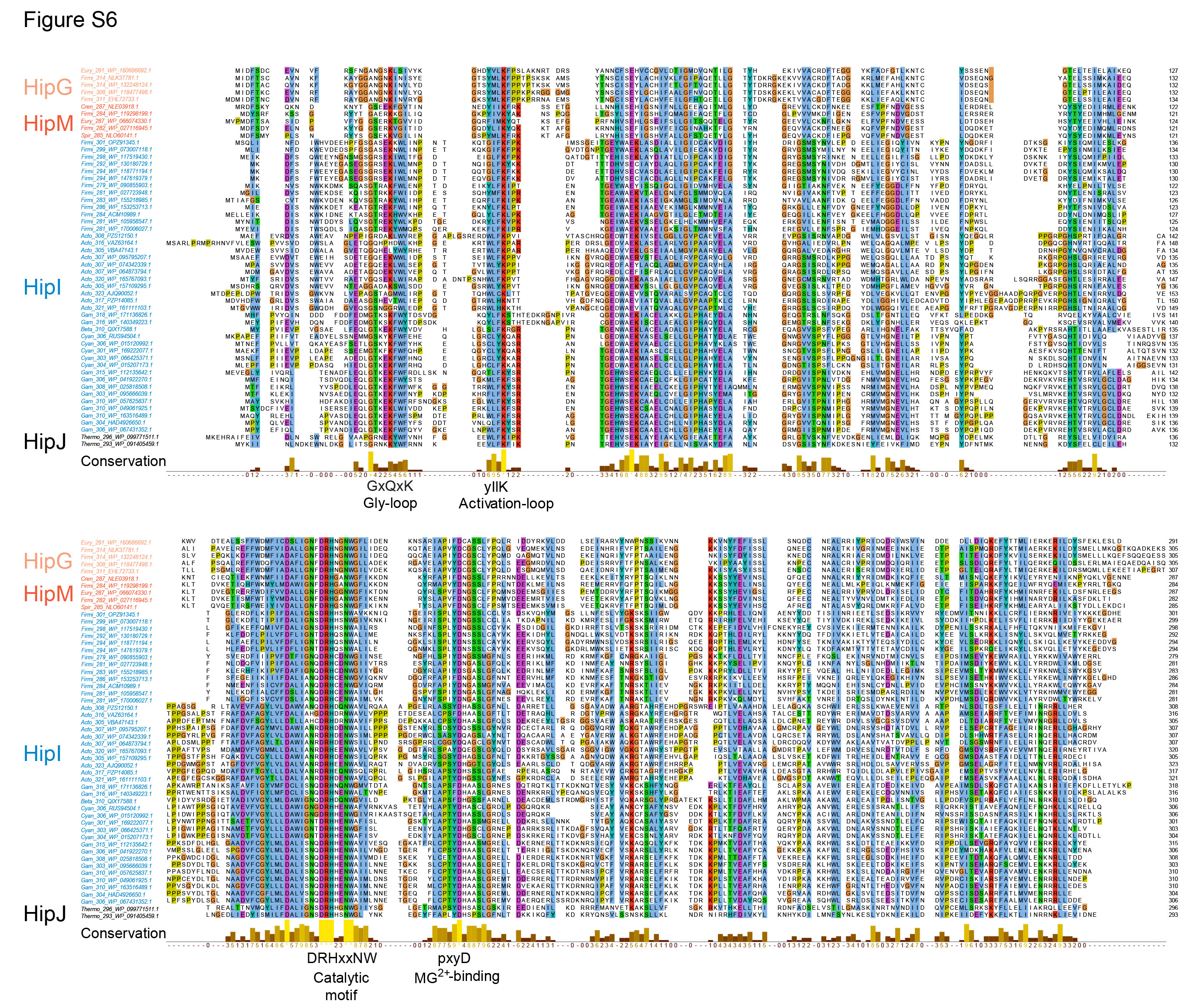

Supplement: FIG S6 [file mbio.01058-21-sf006.jpg]

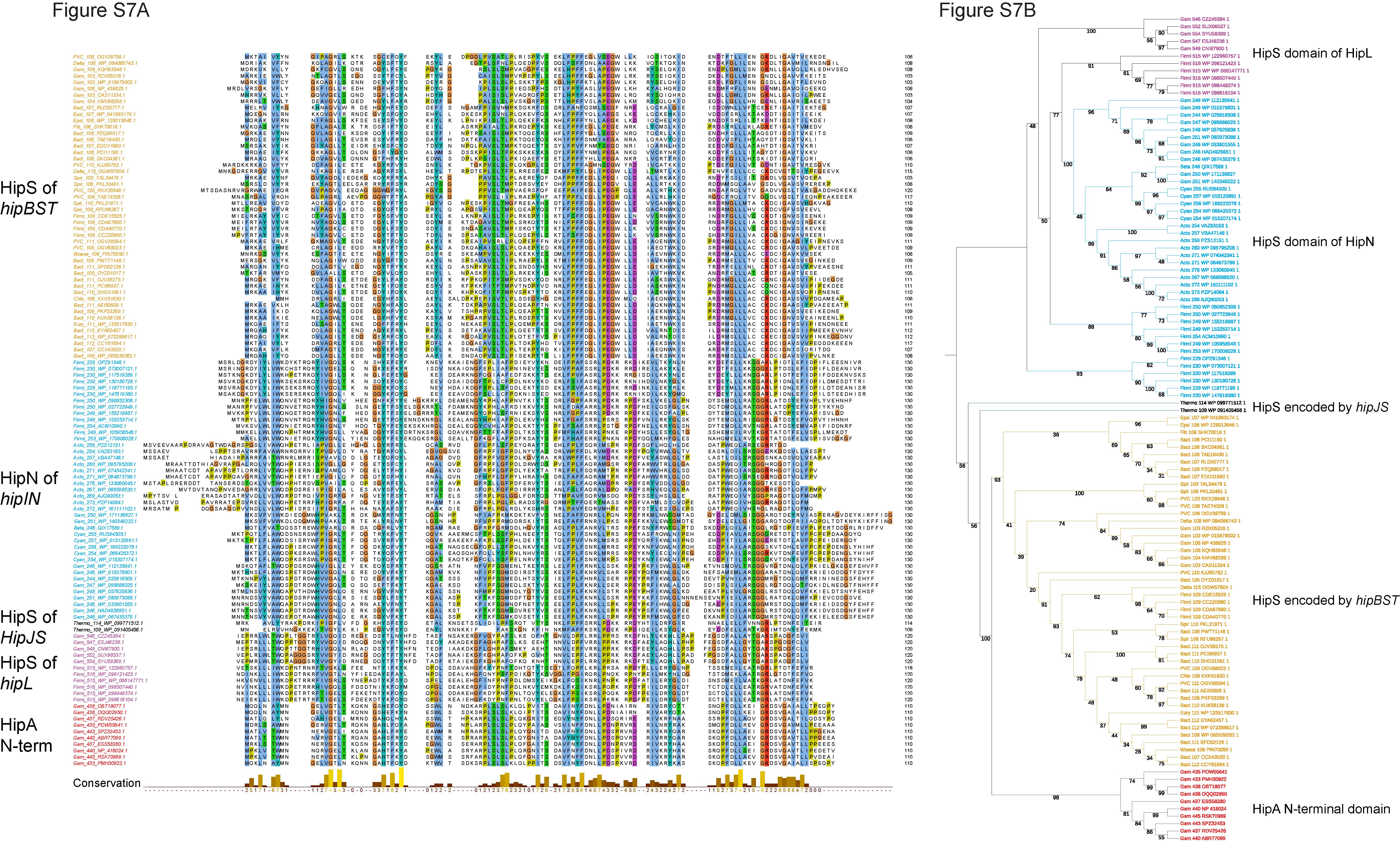

Supplement: FIG S7 [file mbio.01058-21-sf007.jpg]

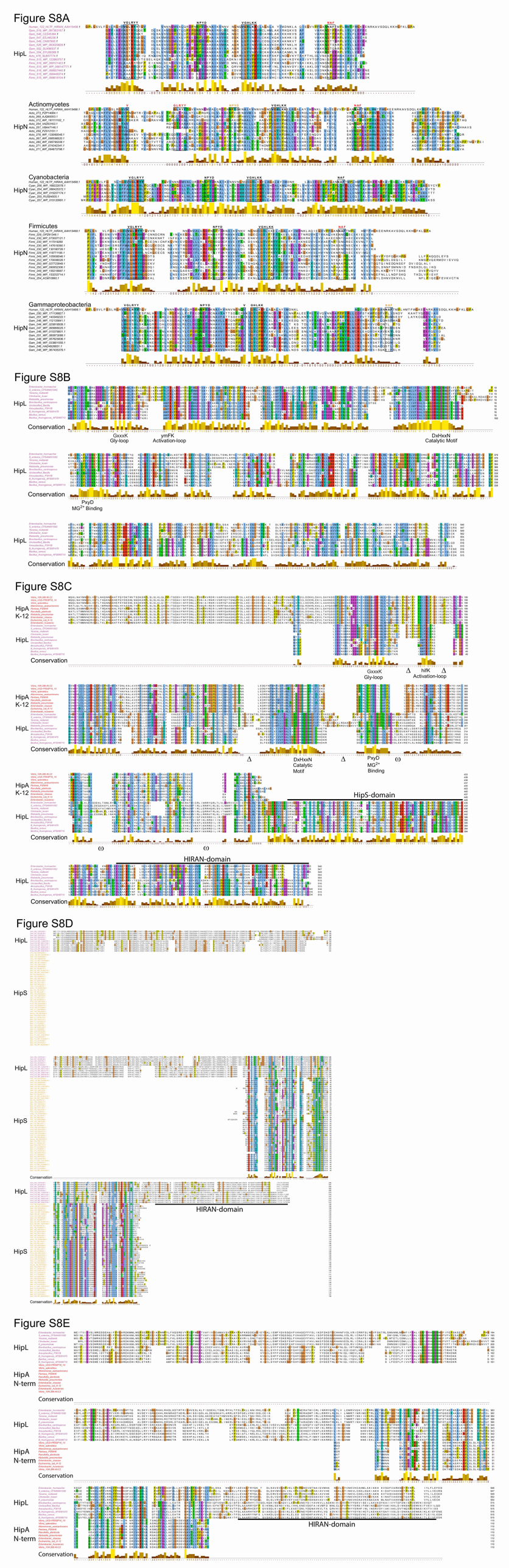

Supplement: FIG S8 [file mbio.01058-21-sf008.jpg]
