# Supplementary material for: Phylogeny Reveals Novel HipA-Homologous Kinase Families and Toxin-Antitoxin Gene Organizations
Source: mBio. 2021 Jun 1;12(3):e01058-21. doi: 10.1128/mBio.01058-21 (PMC8262856; doi:10.1128/mBio.01058-21)
Supplement: FIG S4 [file mbio.01058-21-sf004.pdf]

Figure S4A

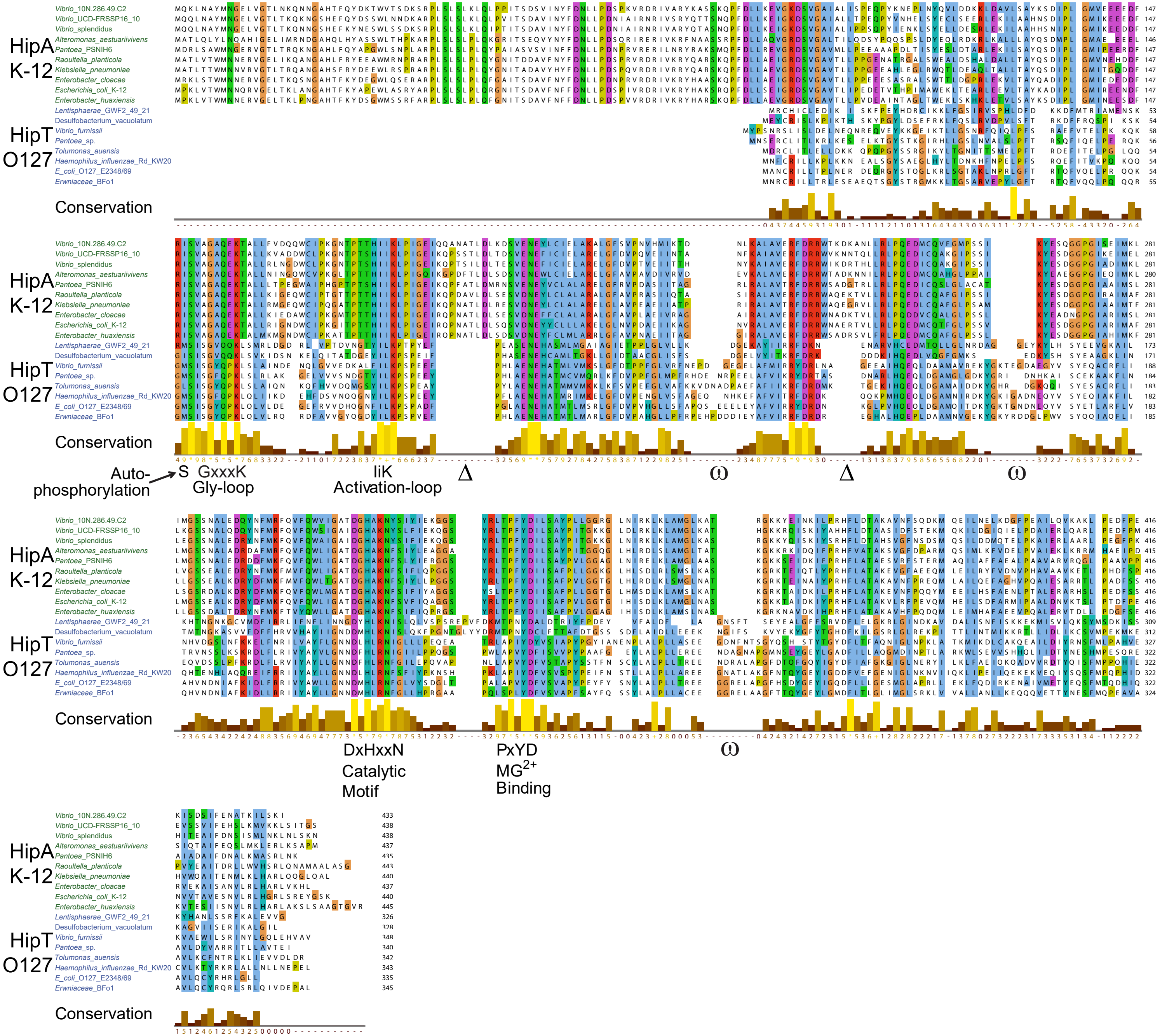

HipA K-12
Vibrio_10N.286.49.C2
Vibrio_UCD-FRSSP16_10
Vibrio_splendidus
Alteromonas_aestuariivivens
Pantoea_PSNIH6
Raoultella_planticola
Klebsiella_pneumoniae
Enterobacter_cloacae
Escherichia_coli_K-12
Enterobacter_huaxiensis
HipT O127
Lentisphaerae_GWF2_49_21
Desulfobacterium_vacuolatum
Vibrio_furnissii
Pantoea_sp.
Tolumonas_auensis
Haemophilus_influenzae_Rd_KW20
E_coli_O127_E2348/69
Erwniaceae_BFo1
Conservation
Auto-phosphorylation
S
GxxxK
Gly-loop
liK
Activation-loop
Δ
ω
DxHxxN
Catalytic Motif
PxYD
MG2+ Binding

Figure S4B

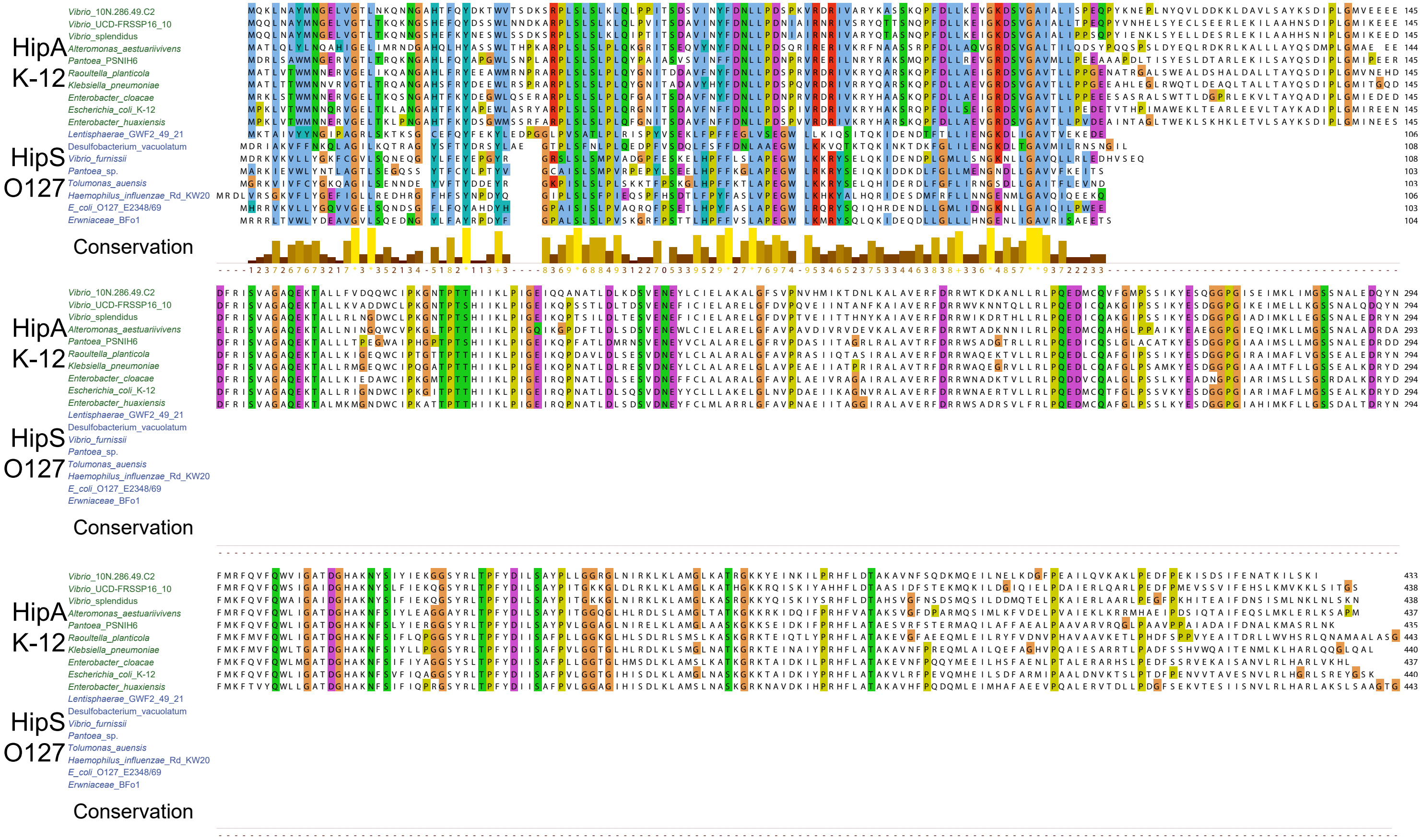

HipA K-12
HipS O127
Vibrio_10N.286.49.C2
Vibrio_UCD-FRSSP16_10
Vibrio_splendidus
Alteromonas_aestuariivivens
Pantoea_PSNIH6
Raoultella_planticola
Klebsiella_pneumoniae
Enterobacter_cloacae
Escherichia_coli_K-12
Enterobacter_huaxiensis
Lentisphaerae_GWF2_49_21
Desulfobacterium_vacuolatum
Vibrio_furnissii
Pantoea_sp.
Tolumonas_auensis
Haemophilus_influenzae_Rd_KW20
E_coli_O127_E2348/69
Erwiniaceae_BFo1
Conservation
