# Supplementary material for: Phylogeny Reveals Novel HipA-Homologous Kinase Families and Toxin-Antitoxin Gene Organizations
Source: mBio. 2021 Jun 1;12(3):e01058-21. doi: 10.1128/mBio.01058-21 (PMC8262856; doi:10.1128/mBio.01058-21)
Supplement: Table S2 [file mbio.01058-21-st002.docx]

| Table S2A. The 1,239 Hip Kinases Divided on Phyla^a)^ | | |  |
| --- | --- | --- | --- |
| Phyla | Phyla Short Names^a)^ | Number of kinases | In % |
| Gammaproteobacteria | Gam | 322 | 26,0 |
| Betaproteobacteria | Beta | 239 | 19,2 |
| Alphaproteobacteria | Alpha | 199 | 16,1 |
| Zetaproteobacteria | Zeta | 4 | 0,32 |
| Deltaproteobacteria | Delta | 76 | 6,1 |
| Epsilonproteobacteria | Epsi | 21 | 1,7 |
| Proteobacteria | Prot | 6 | 0,48 |
| All Proteobacteria | | 867 | 70,0 |
| Nitrospirae | Nitro | 4 | 0,32 |
| Acidobacteria | Acid | 4 | 0,32 |
| Bacteriodetes | Bact | 34 | 2,7 |
| Actinomycetes | Acto | 166 | 13,4 |
| Firmicutes | Firmi | 66 | 5,3 |
| Tenericutes | Tene | 2 | 0,16 |
| Fusobacteria | Fus | 2 | 0,16 |
| Fibrobacter | Fib | 4 | 0,32 |
| Chlamydiales/Verrucomicrobia/Planctomycetes | PVC | 39 | 3,14 |
| Cyanobacteria | Cyan | 6 | 0,48 |
| Chlorobi | Chloro | 1 | 0,08 |
| Spirochaetes | Spir | 23 | 1,9 |
| Aquificae | Aqui | 1 | 0,08 |
| Thermotogae | Thermo | 2 | 0,16 |
| Deferribacteres | Defer | 6 | 0,48 |
| Ignavibacteriae | Igna | 1 | 0,08 |
| Chrysiogenales | Chrys | 1 | 0,08 |
| Crenarchaeota | Cren | 2 | 0,16 |
| Euryarchaeota | Eury | 6 | 0,48 |
| Woesearchaeota | Woese | 2 | 0,16 |
|  |  | 1,239 | 100 |

1. The kinases are listed in **Table S1**
2. Short names of the phyla used in **Table S1**

**Table S2B. Frequencies of gene organizations of the 1,239 Hip Tree**

| Gene Organization | Number of modules^a)^ | In % |
| --- | --- | --- |
| *hipBA* | 989 | 80 |
| *hipAB*^a)^ | 8 | >1 |
| *hipEB* | 12 | 1 |
| *hipBST* | 48 | 4 |
| *hipH* | 101 | 8 |
| *hipL* | 14 | 1 |
| *hipQF* | 14 | 1 |
| *hipQG* | 5 | >1 |
| *hipIN* | 14 | 1 |
| *hipJS* | 2 | >1 |
| *hipMP* | 5 | >1 |
| Total | 1,239 | 100 |

a) Numbers derived from **Table S1**.

b) Two kinase sub-clades, on in Main Clade V and one in Main Clade VII,

have a reversed toxin – antitoxin gene order as compared to that of *hipBA*.

Because these kinase sub-clades are located deep within their respective

Main Clades we decided not to define them as new kinase families.
